# MethylationToActivity: a deep-learning framework that reveals promoter activity landscapes from DNA methylomes in individual tumors

**DOI:** 10.1186/s13059-020-02220-y

**Published:** 2021-01-19

**Authors:** Justin Williams, Beisi Xu, Daniel Putnam, Andrew Thrasher, Chunliang Li, Jun Yang, Xiang Chen

**Affiliations:** 1 Department of Computational Biology, St. Jude Children’s Research Hospital, 262 Danny Thomas Place, Mail Stop 1135, Memphis, TN 38105 USA; 2 Center for Applied Bioinformatics, St. Jude Children’s Research Hospital, Memphis, TN USA; 3 Department of Tumor Cell Biology, St. Jude Children’s Research Hospital, Memphis, TN USA; 4 Department of Surgery, St. Jude Children’s Research Hospital, Memphis, TN USA

**Keywords:** DNA methylation, Histone modifications, Convolutional neural network, Transfer learning

## Abstract

Although genome-wide DNA methylomes have demonstrated their clinical value as reliable biomarkers for tumor detection, subtyping, and classification, their direct biological impacts at the individual gene level remain elusive. Here we present MethylationToActivity (M2A), a machine learning framework that uses convolutional neural networks to infer promoter activities based on H3K4me3 and H3K27ac enrichment, from DNA methylation patterns for individual genes. Using publicly available datasets in real-world test scenarios, we demonstrate that M2A is highly accurate and robust in revealing promoter activity landscapes in various pediatric and adult cancers, including both solid and hematologic malignant neoplasms.

**Supplementary Information:**

The online version contains supplementary material available at 10.1186/s13059-020-02220-y.

## Background

Transcriptional regulation is fundamental to the identity and function of cells. Deregulation of gene expression is a defining feature of common diseases, including cancers. Promoters, the regulatory regions surrounding the transcription starting sites (TSSs), integrate signals from distal enhancers and local histone modifications (HMs) to initiate transcription. Almost half of human protein-coding genes harbor multiple TSSs; consequently, promoter activities determine both the level of transcription and the transcript isoforms that are expressed, with the latter potentially having different translation efficiencies and encoding different protein sequences [1]. Tumors frequently use alternative promoters to increase the isoform diversity [2, 3], to activate oncogenes that are normally repressed [1–3], and to evade host immune attacks by immunoediting [3, 4]. Compared to cancers in adults, pediatric tumors harbor fewer mutations [5, 6] and use epigenetic deregulation to promote tumorigenesis and progression [7].

Promoter activities can be determined experimentally through transcriptomic approaches, such as CAP analysis gene expression (CAGE), or through epigenomic approaches, including chromatin immunoprecipitation followed by sequencing (ChIP-seq) [8]. Because of transcript degradation by 5′ RNA exonucleases, ChIP-seq approaches for specific HMs have been the gold standard for studying promoter activities [9]. Several studies [10–12] have demonstrated that HMs and other epigenetic features can be used to predict gene expression. Using a linear regression model, Karlić et al. show that approximately 50–60% of the variation in gene expression can be accounted for and that ~ 50% of the variation in gene expression can be modeled by promoter H3K27ac enrichment alone [10]. Subsequent work by Dong et al. further explained 69% of gene expression variance using a hybrid random forest/linear regression model with features derived from 11 HMs, one histone variant, and DNase I hypersensitivity [11]. More recently, Singh et al. used deep-learning models on five HMs to predict gene expression status (high/low) and achieved an average AUC of 0.80 [12]. However, the scarcity of pediatric tumors, the limited amounts of fresh starting material available, and the extensive workload involved in acquiring the promoter activity landscapes constrain their interrogation for individual patient tumors [13, 14].

DNA methylation (DNAm) is a well-studied, relatively stable, and inheritable epigenetic regulatory mechanism that involves transferring a methyl group to cytosine (C) to form 5-methylcytosine (5mC), mostly in the CpG context. In contrast to HMs, DNAm can be accurately and robustly profiled in various tissues, including archival formalin-fixed, paraffin-embedded (FFPE) tumor samples, through both array [15, 16] and sequencing [17] platforms; therefore, it has exceptional applicability to studying epigenetic deregulation in tumors. Consequently, genome-wide DNAm profiles represent a widely available epigenetic asset for studying epigenetic abnormalities in primary tumors.

The DNAm pattern is mechanistically connected with transcription factor binding and HMs [18–25]. It also plays critical roles in establishing the chromatin structure in physiologic and pathologic conditions [26, 27]. Moreover, recent applications of machine learning to genome-wide DNAm patterns have demonstrated that DNAm can accurately predict the patterns of chromatin packaging (A/B compartments, the square of the Pearson correlation coefficient *R*^2^ = 0.50–0.66) [28–30] and can reveal distinct subgroups with prognostic significance among patients with cancer [31, 32]. Recently, DNAm signature-based molecular classifiers were shown to improve diagnostic accuracy, as compared to that of traditional schemes, further demonstrating the critical regulatory roles of DNAm in tumor development [33, 34]. However, unlike HMs, where established biological interpretations of various marks have resulted in a general “histone code” hypothesis [35, 36], the relation between DNAm signatures and their transcriptional regulatory roles is complex and nonlinear. In many cases, even promoter DNAm may positively and negatively correlate with gene expression depending on the genomic structure involved in a given tumor [37]. Consequently, with few exceptions (e.g., hypermethylation of the promoters of *RB1*, *CDKN2A*, and *MGMT*) [38], the contribution of DNAm to the regulation of expression of individual genes remains largely elusive [39–41]. Recent attempts to use DNAm signatures to account for gene expression levels have had limited success, with the best model (binomial distribution probit regression [BPR] model) capturing 25–49% of the expression variations [42]. Undoubtedly, the lack of interpretability of the DNAm pattern at the individual gene level has severely hampered our understanding of the biological significance of DNAm signatures.

To address these challenges, we have developed MethylationToActivity (M2A), a deep-learning framework. The central hypothesis of M2A is that the complex relation between DNAm signatures and promoter activities (measured as H3K4me3 and H3K27ac enrichment in the TSS ± 1 kb region) can be captured by incorporating both summary statistics extracted from window-based CpG methylation levels and high-order spatial information from these windows in the promoter and flanking regions (up to 25 kb from the TSS). Using a cohort of six pediatric neuroblastoma (NBL) orthoptic patient-derived xenograft (O-PDX) samples profiled in the Pediatric Cancer Genome Project (PCGP) [43], we trained the model using whole-genome bisulfite sequencing (WGBS) data to predict the enrichment of H3K4me3 and H3K27ac (two HMs critical for promoter [10]) for genome-wide annotated promoters. We validated the predictive accuracy of the model in the remaining NBL samples (*N* = 10, WGBS) from the same cohort. We further confirmed its accuracy and generalizability in diverse tumor types from four publicly available datasets representing real-world applications, including (1) pediatric rhabdomyosarcoma (RMS) O-PDX tumors profiled in the Pediatric Cancer Genome Project (*N* = 16, WGBS) [44]; (2) a set of commonly used cell lines profiled in ENCODE (*N* = 9, WGBS) [45]; (3) primary acute myeloid leukemias (AMLs) profiled by the BLUEPRINT consortium (*N* = 19, WGBS) [46]; and (4) a large primary Ewing sarcoma (EWS) cohort using reduced-representation bisulfite sequencing (RRBS) (*N* = 140) [22]. These applications demonstrate that M2A can accurately reveal promoter activities from DNAm patterns, which will be of great use not only in functionally interpreting differential DNAm patterns but also in profiling promoter usage in individual patient tumors. This will facilitate precision medicine by tailoring treatments based on both genetic variants and epigenetic deregulations.

## Results

### Extensive diversity of promoter activity among *MYCN-*amplified NBL cell lines and O-PDX models

**Fig. 1:**
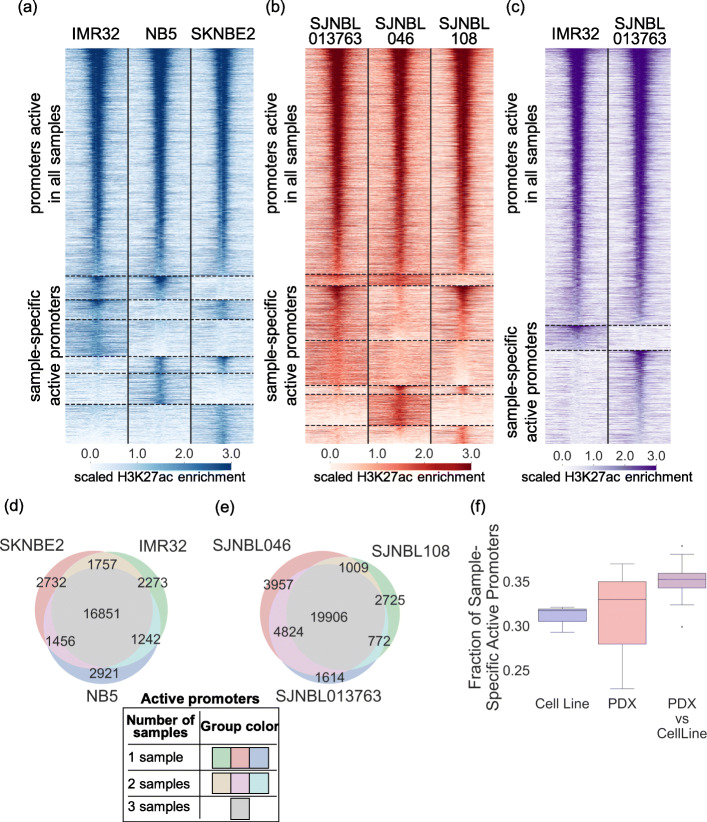
Variations of promoter H3K27ac levels among *MYCN-*amplified NBL samples. **a**–**c** Promoters were classified as active or inactive based on H3K27ac levels in individual NBL tumors: **a** NBL cell line samples, **b** NBL O-PDX samples, and **c** NBL cell line and O-PDX samples. Each promoter region plotted spans TSS ± 5000 bp binned by non-overlapping 250-bp windows; the color bar represents the scaled windowed H3K27ac enrichment, from 0 (lowest) to 3 (highest). Horizontal dotted lines delineate shared promoters (active in all samples) and sample-specific promoters (promoter activity in at least one sample was different from the remaining samples). Promoters were sorted by average descending H3K27ac enrichment across all samples within each group. **d**, **e** Venn diagram indicating the number of shared and sample-specific H3K27ac promoter activities between NBL cell line samples (**d**) and O-PDX samples (**e**). **f** The promoter activity variation is highlighted by the proportion of sample-specific active promoters among all H3K27ac active promoters, as compared within cell line or O-PDX NBL samples, and between cell line and O-PDX NBL samples

To date, most cancer HM profiling studies have made use of tumor models, including cell lines, xenografts, and more recently, organoids. Technical limitations and challenges when working with human tumor tissues prevent the generation of high-quality ChIP-seq profiles for primary patient specimens [47]. Despite the documented epigenetic heterogeneity [48], a common practice in deciphering major HM deregulations in various cancers is to extrapolate the epigenetic profiles from related cancer models (surrogate models). Many studies [43, 44, 49–52] have compared model systems to primary tumors with respect to characteristics such as mutations, gene expression, and DNAm signatures. In this study, we began by evaluating the level of promoter activity diversity in closely related NBL models. Specifically, we evaluated promoter activity, as measured by the H3K27ac level, in three O-PDX models (SJNBL046, SJNBL108, and SJNBL013763) and three cell line models (IMR-32, NB-5, and SKNBE2) that harbor *MYCN* amplification with no other major oncogenic mutations. All samples displayed a bimodal distribution of promoter H3K27ac levels across the genome (Additional file 1: Fig. S1), and O-PDX models had a marginally higher fraction of active promoters (mean 31.9%, range 27.6–36.1%) than did cell line models (mean 26.1%, range 25.6%–26.7%) (*P* = 0.14, Student’s *t* test). However, there were extensive variations in the promoter activities in both the cell line models (Fig. 1a, d) and the O-PDX models (Fig. 1b, e). Moreover, greater divergence was observed between a cell line model and an O-PDX model (mean 34.9%, range 29.9–39.0%) than between two cell line models (mean 31.0%, range 29.2–32.1%; *P* = 0.44, Student’s *t* test) or between two O-PDX models (mean 31.0%, range 22.9–37.0%; *P* = 0.02, Student’s *t* test) (Fig. 1f). Variations in promoter activity may play a significant role in the transcriptional deregulation of individual tumors, as a substantial fraction of established cancer consensus genes (22.4% in O-PDX models and 31.1% in cell line models, including *APOBEC3B*, *TGFBR2*, *PAX7*, *HOXA11*, *PDCD1LG2*, *PTK6*, *BCL11B*, *FAS*, and *MYC*; (Additional file 2: Table S1) displayed heterogeneous promoter activities in the surveyed tumor models. Therefore, we sought to develop a computational approach to infer the promoter activity landscape for individual tumors.

### M2A: a deep-learning framework to reveal promoter activities from DNA methylation

DNAm plays a critical role in determining the framework of gene expression for a given cell/cellular state. However, the highly complex and nonlinear relations between DNAm patterns and HMs severely hamper the interpretability of the biological impact of differential DNAm patterns. Previous studies have shown the usefulness of extracting higher-order methylation features [42], for predicting gene expression. Moreover, recent studies applied deep-learning approaches to infer DNAm states from their local sequence composition and adjacent DNAm states [53]. We hypothesize that these high-level DNAm features (that capture the spatial information from DNAm patterns in the promoter and regions in its vicinity) could also provide an opportunity to infer promoter activities such as H3K27ac and H3K4me3 enrichment accurately. We propose to use a convolutional neural network (CNN)-based deep-learning framework to extract such features.

**Fig. 2:**
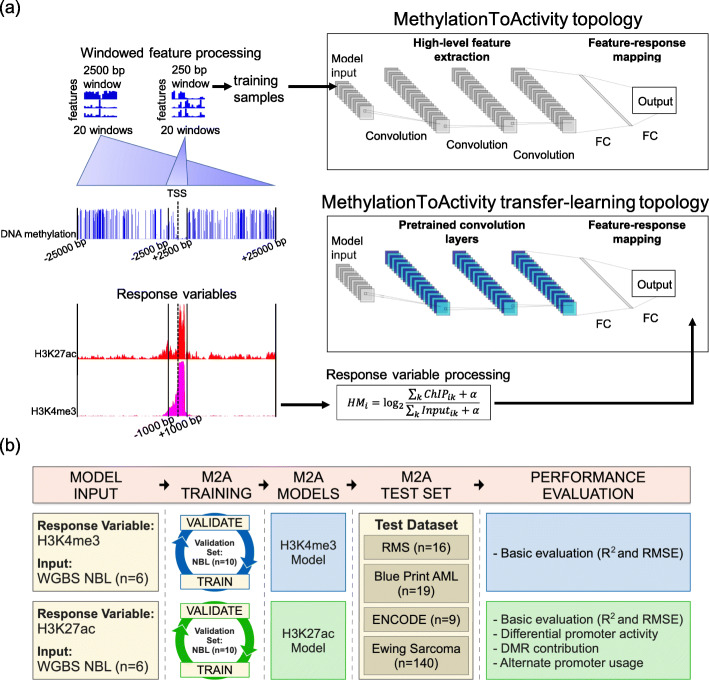
M2A feature processing and training workflow. The M2A framework hinges on the feature processing pipeline. **a** First, windowed features (20 total non-overlapping windows for each of two sizes including 250 bp and 2500 bp) centered around the TSS are calculated from WGBS data for each unique promoter region, extending up to 2500 bp (250 bp window), and 25 kbp (2500 bp window) away from the TSS. Response variables (H3K27ac and H3K4me3) for separate model training were generated for matching promoter regions (TSS ± 1 kbp). The matching window features and response variables serve as input to the model topologies, where M2A first extracts high-level features by using a series of convolutional layers then maps these features to response variables in fully connected (FC) layers. Transfer learning with M2A leverages pretrained feature extraction (frozen CNN layers indicated in blue), training only the FC layers. **b** The overall workflow for training, validation, and testing M2A is detailed, as well as an overview of the analyses performed to validate M2A performance in different real-world applications. M2A models for H3K4me3 and H3K27ac were trained separately, indicated by blue (H3K4me3) and green (H3K27ac)

The M2A conceptual framework and workflow is shown in Fig. 2. M2A starts with raw DNAm feature extraction from around individual TSSs (Fig. 2a). This is followed by high-level feature extraction through the CNN layers and mapping between the generalized feature and the final output (i.e., the H3K4me3 and H3K27ac of the promoter) in the fully connected (FC) layers. The vanilla model described in this report was trained on six NBL PDX tumors (SJNBL046_X, SJNBL013761_X1, SJNBL012401_X1, SJNBL013762_X1, SJNBL013763_X1, and SJNBL015724_X1; Fig. 2b) for which comprehensive genomic and epigenomic profiling data are available, including the results of whole-genome sequencing, whole-exome sequencing, RNA sequencing, WGBS, and ChIP-seq of eight histone marks (H3K4me1, H3K4me2, H3K4me3, H3K27me3, H3K27ac, H3K36me3, H3K9/14 ac, and H3K9me3), CTCF, BRD4, and RNA polymerase II (PolII).

We started with an analysis of the information content in DNAm patterns by examining the input feature distribution in different windows, among active (high H3K27ac), poised (high H3K4me3 and low H3K27ac), and inactive promoters (low H3K4me3 and low H3K27ac) in the six NBL O-PDX training samples. These features show distinct patterns among the three promoter categories (Additional File 1: Fig. S2), indicating the feasibility of modeling promoter activities from DNAm patterns. Although the interpretability of CNN-extracted features remains an active field of research in deep-learning [54], we examined the efficacy of CNN-extracted features in modeling the promoter activities. We first compared the square of Pearson’s correlation (*R*^*2*^) between each feature (both raw input and CNN-extracted features) and the response variable (H3K27ac) in the training set, The analysis revealed that CNN-extracted features have significantly higher *R*^*2*^ with the response (250 bp: *P* = 1.5 × 10^− 11^, 2500 bp *P* = 3.9 × 10^− 5^, Wilcoxon signed-rank test, Additional File 1: Fig. S3). We further evaluated the best features for both raw input and CNN-extracted features in the validation samples and again the CNN-extracted features significantly outperformed the raw input features (250 bp: *P* = 1.1 × 10^− 5^, 2500 bp *P* = 1.1 × 10^− 5^, Wilcoxon signed-rank test, Additional File 1: Fig. S3).

### M2A produces a highly accurate landscape of promoter activity in pediatric NBL

**Fig. 3:**
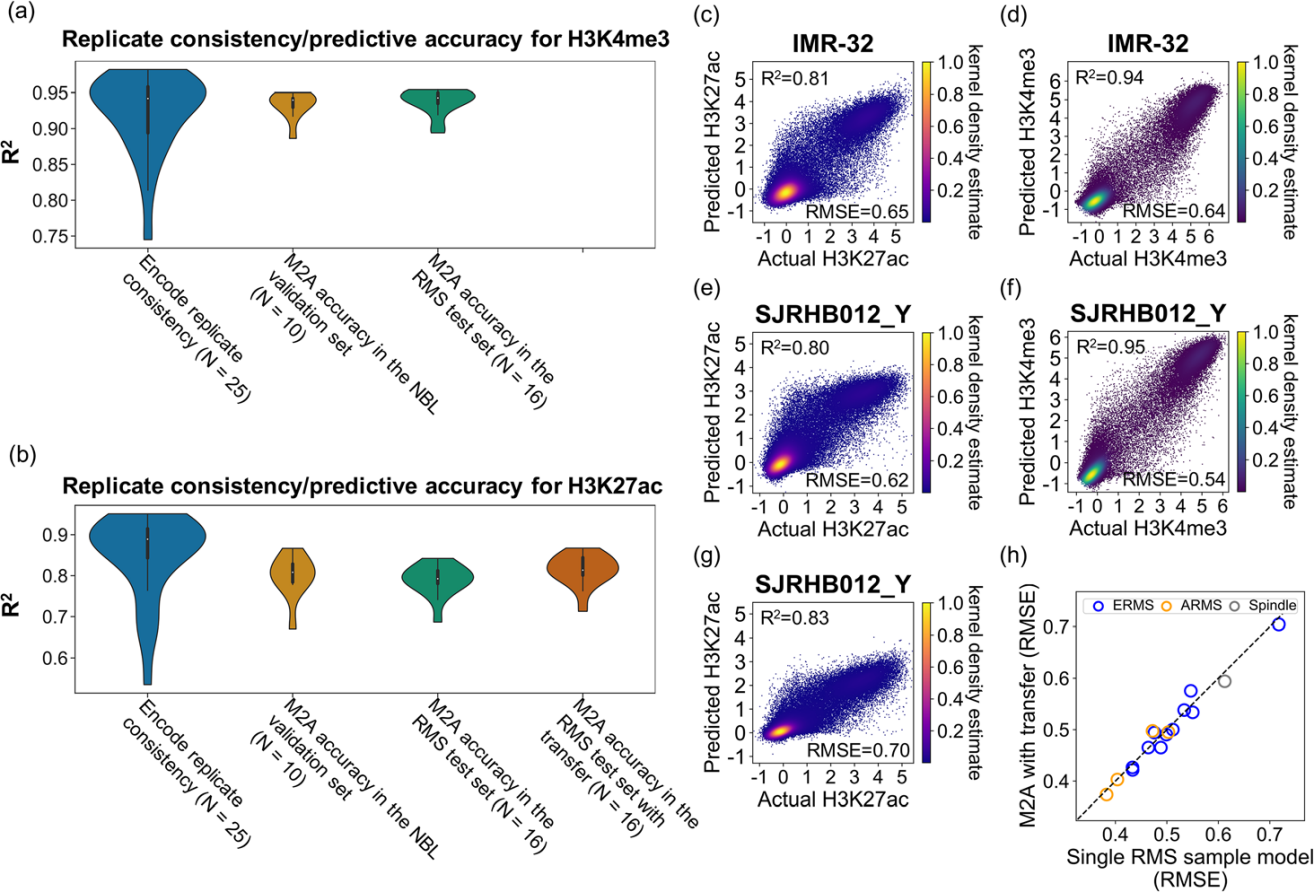
M2A performance in NBL and RMS cohorts. **a, b** Analysis of the performance of M2A in NBL and RMS cohorts with **a** H3K4me3 inference and **b** H3K27ac inference. ENCODE replicate consistencies were calculated as Pearson’s correlation squared (*R*^*2*^) between replicates (two replicates per sample). ENCODE sample KMS-11 was excluded as an apparent outlier *R*^*2*^ = 0.016, RMSE = 1.869. Prediction accuracy was measured by *R*^*2*^ between the actual measurement and M2A’s prediction. **c–g** Individual examples of median M2A performers in **c** NBL cell line H3K27ac inference, **d** NBL cell line H3K4me3 inference, **e** RMS H3K27ac inference (pre-transfer), **f** RMS H3K4me3 inference, and **g** RMS H3K27ac inference (post-transfer). To indicate the density of data points where individual data points cannot be resolved, a KDE was applied, called from 1 (highest) to 0 (lowest). **h** The boost to M2A performance (measured by RMSE) due to transfer learning is shown, as applied and tested in RMS samples

To evaluate the performance of M2A, we first explored its performance in the remaining NBL samples in the cohort (the validation set), including one O-PDX tumor, one primary autopsy tumor, and eight cell lines. Using the validation set, we compared the performance of the M2A framework of three CNN layers and two FC layers (Fig. 2) with three frequently used statistical and machine learning approaches (baseline models), namely multivariate adaptive regression splines (MARS), random forest, and artificial neural network (ANN) consisting of only two FC layers. In every instance, the M2A framework outperformed baseline models (Additional file 2: Table S2). From a qualitative perspective, M2A correctly revealed the bimodal distribution of the promoter activities for both H3K4me3 and H3K27ac in all samples, and from a quantitative perspective, the inferred genome-wide promoter activity landscape was highly accurate for individual samples for both H3K4me3 (*R*^2^ = 0.933 ± 0.019; RMSE = 0.621 ± 0.072) (Fig. 3a, d) and H3K27ac (*R*^2^ = 0.799 ± 0.053; RMSE = 0.644 ± 0.074) (Fig. 3b, c). Moreover, the addition of CNN layers was merited, as there was a decrease in the prediction error (measured as 1 − *R*^2^) from the next highest performer by 17.8% (*P* = 0.0020, Wilcoxon signed-rank test) and 12.4% (*P* = 0.0020, Wilcoxon signed-rank test) for the model topologies for H3K4me3 and H3K27ac, respectively (Additional file 2: Table S2).

Our analysis of *MYCN*-amplified NBL cell line and O-PDX models has revealed substantial variations in their promoter activities, which is a potential caveat to the practice of surrogate model (representing primary tumor epigenomes by a few profiled models). Conversely, M2A produced highly accurate promoter activity landscapes, significantly outperforming the observed consistency between training and testing samples for both H3K4me3 (*R*^2^ = 0.891 ± 0.023, *P* = 2.3 × 10^− 5^, Wilcoxon rank-sum test) and H3K27ac (*R*^2^ = 0.720 ± 0.045, *P* = 9.5 × 10^− 5^, Wilcoxon rank-sum test; Additional file 2: Table S3). Remarkably, in nine (of 10) test samples, the accuracy of the M2A-inferred promoter H3K27ac activity was better than the highest similarity attained by any individual training sample (*P* = 0.027, Wilcoxon signed-rank test). The same pattern was observed for H3K4me3 levels, with M2A being more accurate for nine of 10 samples (*P* = 0.037, Wilcoxon signed-rank test), demonstrating the accuracy of M2A in revealing individual tumor promoter activity landscapes. Finally, the predictive accuracy of M2A was comparable to the experimental consistency observed between replicates from the same cell lines profiled in ENCODE for H3K4me3 (*R*^2^ = 0.933 ± 0.018 for M2A vs. 0.922 ± 0.056 for ENCODE replicates [*N* = 25]; *P* = 0.55, Wilcoxon rank-sum test) (Fig. 3a; Additional file 2: Table S4). The accuracy of M2A also approached the replicate consistency for H3K27ac (*R*^2^ = 0.799 ± 0.050 for M2A vs. 0.849 ± 0.047 for ENCODE replicates [*N* = 26]; *P* = 0.0078, Wilcoxon rank-sum test) (Fig. 3b: Additional file 2: Table S4). Measurement of the root mean square error (RMSE) revealed a similar pattern (Additional file 2: Table S4).

### M2A is generalizable and scalable

Aside from the model accuracy, there are two additional requirements with practical importance for deploying a machine learning model (such as M2A) in real-world applications: (1) generalizability, i.e., M2A needs to achieve a similar performance with a set of unseen test samples, including tumor/tissue types not used in the model training, and (2) scalability, i.e., M2A must be able to be applied efficiently to external data.

We first demonstrated the accuracy, generalizability, and scalability of M2A by using test samples from rhabdomyosarcoma (RMS) O-PDX tumors. The RMS O-PDX dataset consists of 16 pediatric RMS tumors (11 embryonal, four alveolar, and one spindle subtype, termed ERMS, ARMS, and spindle subtypes, respectively). As with the NBL cohort, each RMS sample was extensively profiled, including by WGBS, RNA-seq, and ChIP-seq of H3K4me3 and H3K27ac. Using the vanilla M2A model (the 3CNN-FC model trained on the six NBL PDX samples), M2A achieved an overall predictive accuracy with the RMS dataset that was comparable to that of the NBL test group for both H3K4me3 (*R*^2^ = 0.937 ± 0.017, *P* = 0.30; RMSE = 0.639 ± 0.119, *P* = 0.82, Wilcoxon rank-sum test) (Fig. 3a, f; Additional file 2: Table S5) and H3K27ac (*R*^2^ = 0.790 ± 0.037, *P* = 0.44; RMSE = 0.589 ± 0.084, *P* = 0.058, Wilcoxon rank-sum test) (Fig. 3b, e; Additional file 2: Table S5), which was comparable to or significantly outperformed the observed similarities between two different RMS tumors for H3K4me3 (*R*^2^ = 0.917 ± 0.028, *P* = 0.0020; RMSE = 0.646 ± 0.133, *P* = 0.64, Wilcoxon rank-sum test) and H3K27ac (*R*^2^ = 0.780 ± 0.066, *P* = 0.43; RMSE = 0.550 ± 0.095, *P* = 0.14, Wilcoxon rank-sum test). The accuracy of the inferred H3K4me3 activity was comparable to the inter-replicate consistency of the ENCODE samples (*P* = 0.83, Wilcoxon rank-sum test).

By definition, generalizability can be achieved only in the absence of over-fitting (or “memorization” of the training data). Neural networks often fall victim to this problem through a combination of factors, including relatively small training datasets and/or over-parameterization. The relatively consistent expression of housekeeping genes across different tissues may lead to an inaccurate (often inflated) interpretation of the performance measurement in such a model, as evidenced by the relatively high *R*^2^ value (0.663 ± 0.040) between the promoter H3K27ac level of a random RMS test tumor and the most similar NBL training tumor (Additional file 1: Fig. S4a). Therefore, we focused on the set of genes that are differentially expressed (DE) in RMS and NBL PDX samples [51], for which an over-fitted or memorized model would perform poorly. Not surprisingly, the average correlative consistency between the NBL validation samples and the most similar NBL training sample dropped from 0.755 to 0.599 when the measurement was restricted to promoters encoding the DE genes (Additional file 1: Fig. S4b), whereas a sharp decline (from 0.663 to 0.259) was also observed for RMS test tumors (Additional file 1: Fig. S4b). Conversely, the six-PDX NBL-trained M2A model maintained high accuracy for promoters of DE genes in both the NBL validation set (*R*^2^ = 0.729 ± 0.071) and the RMS test set (*R*^2^ = 0.715 ± 0.044) (Additional file 1: Fig. S4b), further demonstrating the generalizability of M2A.

M2A is efficient and scalable. For a local implementation of M2A (source code, built models and a Docker image available at https://github.com/chenlab-sj/M2A), the training of the vanilla M2A model (with six NBL O-PDX tumors) takes approximately 16 min (using a Tesla P100-16GB GPU). The feature extraction and promoter activity prediction from WGBS data (as a genome-wide DNAm level file in a tab-delimited text format) can be executed on a personal computer (in this case, we used a MacBook Air with a 2.2-GHz Intel Core i7 and 8-GB 1600-MHz DDR3 RAM) and takes 15–19 min. Moreover, we have implemented a cloud version of M2A (https://platform.stjude.cloud/workflows/methylation-to-activity), available to the general research community.

### Transfer learning further improves the performance of M2A with minimal additional input in the target domain

Although we have demonstrated the generalizability of M2A in the RMS dataset, the fact that epigenetic genes are frequently mutated in pediatric tumors [7] raises the possibility that individual tumor types carry a type-specific interpretation of the DNAm patterns. When ChIP-seq measurement is available for sufficient samples, a type-specific model is desirable. However, although pediatric solid tumors as a group constitute a rare disease, they comprise many different tumor types, and it is rare to have sufficiently profiled samples available for many of them. In addressing this challenge, we hypothesize that a fixed feature-extraction strategy (transfer learning) can achieve the goal of deriving an efficient tumor type-specific model by using a small labeled dataset. A primary assumption here is that generalized features extracted based on a large dataset are similarly informative for apparently different tasks. The feature learning and selection characteristics of CNNs provide exceptional portability in various tasks with extremely small labeled datasets.

In M2A, the CNN layers capture generalized DNAm features and the FC layers learn the mapping function between the DNAm features and the promoter activities. Here we start with the pretrained vanilla M2A model, fix the feature-extraction layers (CNN layers), and use a single sample from the target tumor type to update the mapping function (the weights and biases of the FC layers). Because the consistency of M2A for H3K4me3 approached the inter-replicate consistency in both NBL and RMS datasets, we focused on H3K27ac inference for transfer learning. Upon performing transfer learning with a single sample in the RMS dataset, we observed significantly improved accuracy (*R*^2^ = 0.813 ± 0.038, *P* = 3.1 × 10^− 5^, Wilcoxon signed-rank test) (Fig. 3b, g, and h; Additional file 2: Table S5). Moreover, this model significantly outperformed a single RMS sample model with the identical model architecture, in which both the CNN layer and the FC layers were derived from the RMS training sample (*P* = 9.2 × 10^− 5^, Wilcoxon signed-rank test) (Additional file 1: Fig. S5; Additional file 2: Table S6) and marginally outperformed the observed similarities between different RMS tumors (*P* = 0.053, Wilcoxon rank-sum test). This analysis demonstrated the value of both the pretrained CNN layers for general feature extraction and a single profiled sample in the target domain. Consequently, we applied transfer learning to both the EWS and AML datasets. However, transfer was not feasible in the ENCODE dataset because those cell lines were derived from different tissues.

### M2A accurately reveals promoter activity landscapes in adult tumors and in hematologic malignant neoplasms

We next evaluated the performance of M2A in independently collected datasets, including ones for adult tumors and hematologic malignant neoplasms. Upon analyzing nine ENCODE cell lines, we found that differences in antibody usage and experimental protocols between the ENCODE and NBL datasets resulted in different signal-to-noise profiles (Additional file 1: Fig. S6), with higher RMSE values between the model predictions and the actual observations (H3K4me3: 0.961 ± 0.238, *P* = .0019; H3K27ac: 0.918 ± 0.188, *P* = 2.6 × 10^− 4^, Wilcoxon rank-sum test). However, the predicted activities remained highly correlated with the experimental measurements (H3K4me3: *R*^2^ = 0.895 ± 0.027; H3K27ac: *R*^2^ = 0.680 ± 0.149) (Additional file 1: Fig. S7). Although the accuracy of H3K4me3 was relatively uniform, H1-ESC and SK-N-SH were outliers with a substantially lower accuracy of H3K27ac inference (by the boxplot-based method [55]) (Additional file 2: Table S4; Additional file 1; Fig. S8a). An investigation of the promoter activity (the measured and inferred H3K27ac levels) and the measured gene expression in H1-ESC revealed that a subset of actively transcribed genes showed little or no H3K27ac levels in their promoters, where M2A inferred relatively strong promoter activity (Additional file 1: Fig. S8b). Consequently, the inference of promoter activity by M2A outperformed the actual measurement in terms of both the quantitative consistency with the gene expression level (*R*^2^ = 0.536 for the M2A-inferred H3K27ac level vs. 0.435 for the measured H3K27ac level) (Additional file 1: Figs. S8b and S8c) and the accuracy in predicting expressed genes (AUC = 0.891 for the M2A-inferred H3K27ac level and 0.881 for observed H3K27ac level) (Additional file 1: Fig. S9d). We also observed a small fraction of inferred active promoters without strong expression; these may represent genes subject to transcriptional pausing (where transcription is initiated but there is no elongation), a distinctive feature of undifferentiated stem cells [56]. Similarly, better consistency with gene expression levels was observed in SK-N-SH (Additional file 2: Table S7).

We further evaluated the performance of M2A in revealing promoter activities in 19 acute myeloid leukemia (AML) primary patient samples collected by the BLUEPRINT consortium, which is part of the International Human Epigenome Consortium (IHEC) [46]. Analyses of observed promoter activity (measured by H3K27ac level) and gene expression (measured in fragments per kilobase of transcript per million mapped reads [FPKM]) in the same sample revealed non-uniform qualities with a wide range of consistency (mean *R*^2^ = 0.539, range 0.178–0.720) (Additional file 2: Table S8). Similarly, the M2A-inferred promoter activity landscape displayed substantial variability with respect to the observed activities among these samples (mean *R*^2^ = 0.473, range 0.031–0.729). Strikingly, the consistency between the observed promoter activity and gene expression was highly predictive of the performance of M2A with individual samples (*R*^2^ = 0.975, *P* = 4.1 × 10^− 15^, Pearson’s correlation test) (Additional file 1: Fig. S10). Finally, although gene expression was not used in model generation with M2A (for the vanilla model or the transfer learning step), the promoter activity inferred by M2A showed uniform consistency with gene expression (mean *R*^2^ = 0.628, range 0.541–0.684) (Additional file 2: Table S8). These results jointly suggest that the ChIP-seq library quality is a potential confounding factor for both the consistency between the observed (from ChIP-seq) and the M2A-inferred promoter activity landscapes and the consistency between the observed promoter activity and gene expression in these samples.

### Promoter activity landscape inferred by M2A faithfully recapitulates the subtype difference between embryonal and alveolar rhabdomyosarcomas

**Fig. 4:**
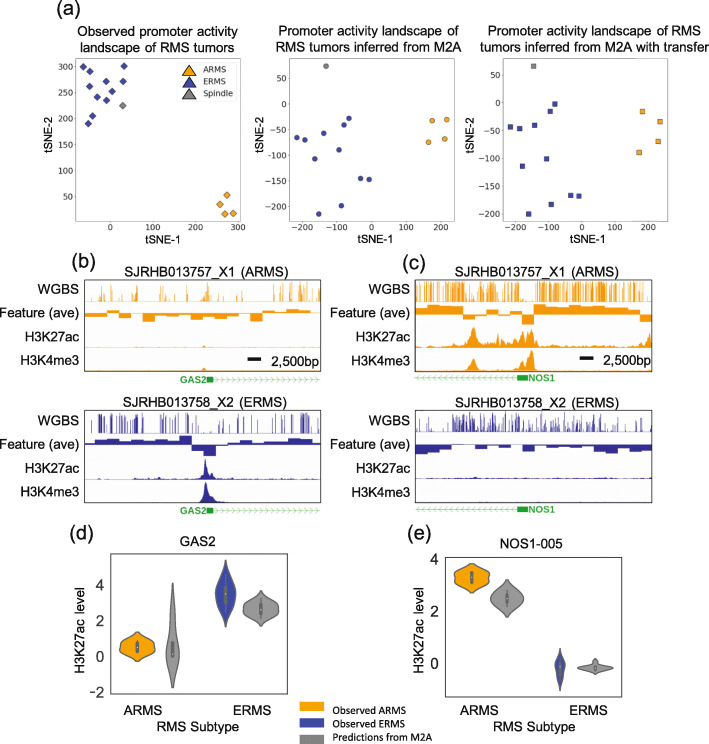
M2A recapitulates subtype differences in RMS. **a** A *t*-distributed Stochastic Neighbor Embedding (tSNE) analysis of observed (left), M2A inferred (center: pre-transfer, right: post-transfer) H3K27ac promoter levels. Embryonal and alveolar RMS subtypes are well separated in each analysis, demonstrating that M2A inferred H3K27ac levels maintain the delineation of RMS subtypes, consistent with the observed H3K27ac tSNE analysis. **b**–**d** The subtype-specific genes *GAS2* (**b**) and *NOS1* (**c**) show subtype distinct patterns of DNAm, H3K27ac, and H3K4me3 levels. The windowed average DNAm feature (2500-bp windows, over the genomic region TSS ± 25 kb) is shown as example (partial) M2A input. These subtype differences were faithfully recapitulated by the M2A H3K27ac inferences for *GAS2* (**d**) and *NOS1* (**e**)

Identifying recurrent epigenetic deregulations (epi-drivers) is a primary research focus in cancer epigenome studies [57]. To this end, we investigated whether subtype-specific epigenetic deregulation was captured in M2A-revealed promoter landscapes in the RMS O-PDX tumors. A tSNE embedding using M2A-inferred promoter activity landscapes (from the NBL-trained model) recapitulated the clear separation of ARMS and ERMS tumors (Fig. 4a) in the DNAm profiles (data not shown), which further demonstrates the generalizability of the CNN-extracted high-order DNAm features. Importantly, when focusing on the promoters of DE genes in the ARMS and ERMS subtypes, the vanilla M2A model faithfully retained the subtype-specific promoter activity patterns (*R*^2^ = 0.713 for DE genes with a single annotated promoter; 0.621 when all annotated promoters for DE genes were included) (Additional file 1: Figs. S11a and S11c). Transfer learning using data from a single RMS further improved the consistency (*R*^2^ = 0.758 and 0.673, respectively) (Additional file 1: Figs. S11b and S11d).

*GAS2* is a gene selectively expressed in ERMS [44]. Although promoter hypomethylation was found in the ARMS tumors, the M2A model correctly predicted significantly stronger promoter activities in ERMS tumors (*P* = 0.01, Wilcoxon rank-sum test) (Fig. 4b, d). Similarly, although both ERMS and ARMS tumors had *NOS1-005* promoter hypomethylation, strong promoter activity was predicted in ARMS tumors only (*P* = 0.0015, Wilcoxon rank-sum test) (Fig. 4c, e), consistent with the ChIP-seq measurement.

### M2A reveals the contribution of differentially methylated regions to promoter activities

Although DNA methylation patterns, including differentially methylated regions (DMRs), are well-established biomarkers for diverse diseases and have revealed molecularly and clinically different subtypes for many cancers, their functional importance in individual gene regulation is less clear [39–41]. For example, many cancer-specific CpG island hypermethylation regions occur in genes that normally are not expressed or are expressed at only a low level [58]. Moreover, even in genes that are both differentially expressed and differentially methylated in different subtypes, upregulated samples can be associated with hypomethylation, hypermethylation, or both [37, 44]. The observed nonlinear relation between DNA methylation and gene expression complicated the functional interpretation of specific DMRs. In this analysis, we interpret the functional roles of DMRs based on the promoter activities of their associated DE genes.

To summarize unambiguously the contribution of DMRs to differential promoter activities, we focused on 371 genes in ERMS and ARMS that have a single annotated promoter and that are both differentially expressed (197 are overexpressed in ERMS, 172 are overexpressed in ARMS) and differentially methylated (169 are hypomethylated, 128 are hypermethylated, and 74 have both hypomethylation and hypermethylation) in the two major RMS subtypes (Additional file 2: Tables S9 and S10) [44]. Among these genes, 140 promoters showed significantly higher H3K27ac measurements in the overexpressed subtype (FDR < 0.1, Wilcoxon rank-sum test), whereas 206 promoters had measurements that were significantly higher when the DNAm-based H3K27ac activity was measured. These 206 promoters included 118 of the 140 promoters identified using the observed signals. These results suggest that M2A can reveal the role of DMRs in modulating the promoter activities of affected genes in a context-specific manner.

### M2A identifies subtype-specific promoter usage encoding different protein isoforms in rhabdomyosarcoma

Alternative promoter usage is an important pretranslational mechanism for tissue-specific regulation as it affects the diversity of isoforms available. Recently, light was shed on the pervasiveness of alternative promoter usage in cancer; in some cases, promoter usage is a more accurate reflection of patient survival than is gene expression [2]. Among 10,835 active genes with multiple annotated promoters in the RMS dataset, we found 2584 genes (24%) with alternative primary promoter usage among 16 samples. We focused on 562 genes that (1) were active in both the ERMS and ARMS subtypes and (2) had subtype-specific promoter usage. We explored the accuracy of M2A in predicting alternative promoter usage in ARMS and ERMS (Additional file 2: Table S11).

Based on measured promoter activities, 428 genes exhibited significant usage difference between the two subtypes (FDR < 0.1, Wilcoxon rank-sum test [used as the ground truth]). The M2A-inferred promoter activity landscape revealed 276 genes for which there was a significant difference in promoter usage between the subtypes (FDR < 0.1, Wilcoxon rank-sum test), and 210 of them matched the ground truth (precision = 0.76, recall = 0.49, F1 score = 0.60) (Additional file 2: Table S11).

**Fig. 5:**
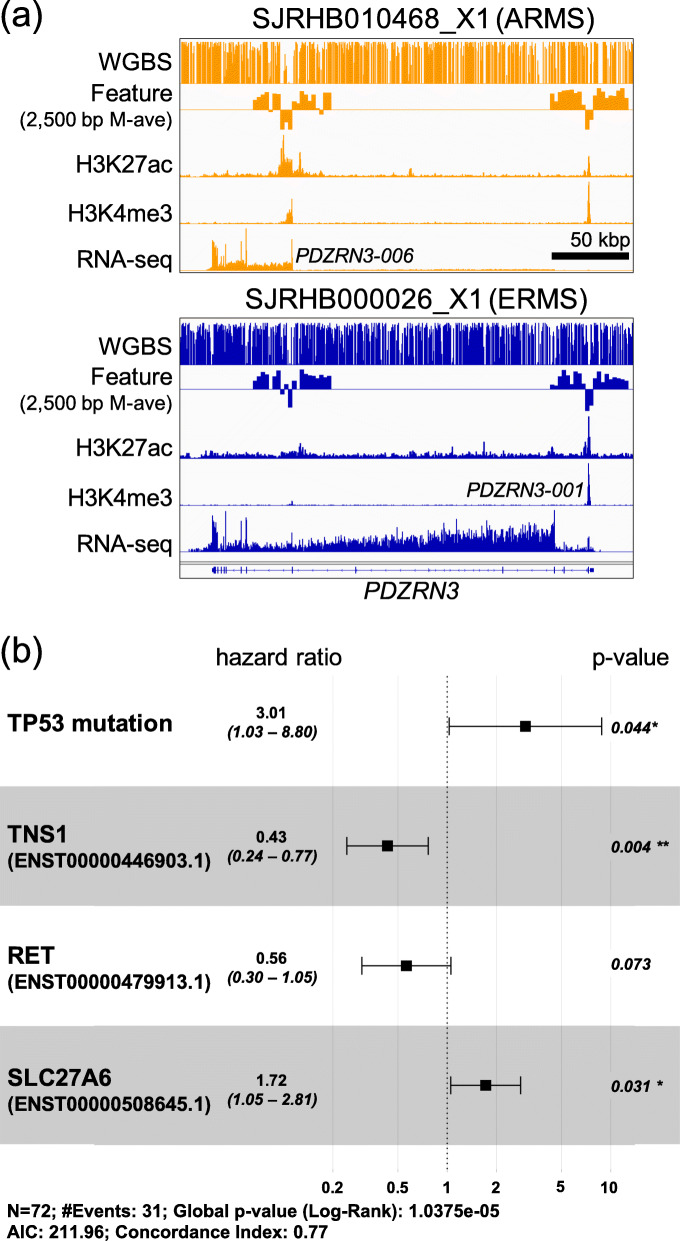
M2A reveals alternate promoter usage in RMS and EWS. **a** An analysis of alternate primary promoter usage between RMS subtypes ERMS and ARMS shows that M2A appropriately predicts subtype-specific promoter usage in *PDZRN3*, a known target of the PAX3/7–FOXO1 fusion protein, which is consistent at the observed values of H3K27ac, H3K4me3, RNA-seq, and DNA methylation. Partial M2A input (DNAm 2500 bp windowed average) is shown to emphasize the DNAm patterns in the genomic region surrounding the TSS ± 25 kb. **b** Alternate promoter usage in EWS patient samples with and without *TP53* mutations were incorporated in a Cox proportional hazards model, highlighting the potential prognostic value of the isoforms identified by M2A

PDZ Domain Containing Ring Finger 3 (*PDZRN3*) is a known target of the PAX3/7–FOXO1 fusion protein [59–61], which blocks terminal differentiation in myogenesis [62]. M2A predicted a subtype-specific promoter usage pattern in *PDZRN3*. Functional studies have shown that PDZRN3 regulates myoblast differentiation into myotubes through transcriptional and posttranslational regulation of Id2 [62]. Its overexpression in ARMS was primarily driven by the fusion protein binding adjacent to an alternative promoter (*PDZRN3-006*) located 191 kbp downstream of the canonical promoter (*PDZRN3-001*) (Fig. 5a). The subtype-specific isoform usage is accompanied by DMRs of the alternative promoter and its immediate downstream regions and is further confirmed by RNA-seq read alignment (Fig. 5a). Compared to the canonical isoform expressed in ERMS, the ARMS-preferred *PDZRN3-006* isoform lacks the RING-finger and Sina domains in the N-terminus and harbors a shorter PDZ domain. The isoform difference, as well as the differences in expression level, between subtypes, may contribute to the impairment of myogenesis at different stages in the development of ARMS and ERMS tumors.

### M2A identifies alternative promoter usages with potential prognostic values in Ewing sarcoma

We next examined the predicted epigenetic promoter activities in 140 EWS samples with DNAm data assayed by RRBS [22]. Three of these samples had matching ChIP-seq profiles for H3K4me3 and H3K27ac. To interrogate this dataset, we applied the pretrained vanilla model with transfer learning, as detailed above, to recalibrate the weights mapping the high-level features to the promoter activities in the EWS cohort. Despite the difference in DNAm platforms (WGBS for the NBL training model and RRBS for the EWS samples), the inferred promoter activity landscape was accurate (*R*^2^ = 0.718, 0.628, and 0.702 for the three samples with HM profiles, using leave-one-out prediction) (Additional file 2: Table S12).

Ewing sarcomas with mutations in *TP53* and *STAG2* have a particularly dismal prognosis [63]. We explored whether the promoter activity had additional prognostic value in 72 samples for which survival data was available. Because of the limited sample size, the initial analysis revealed a significant association between poor clinical outcomes and *TP53* mutations (*P =* 0.00047, log-rank test) (Additional file 1: Fig. S12a) but not *STAG2* mutations (*P* = 0.67, log-rank test) (Additional file 1: Fig. S12b). We identified 21 active genes that showed a potential difference (absolute mean difference of log-scaled activity ≥ 1) between *TP53* mutant tumors and wild-type tumors, and we applied Cox proportional hazards models to evaluate their potential contributions to patient survival that are independent of *TP53* or *STAG2* mutation status (Additional file 2: Table S13). We performed the same analysis for 45 genes with potential different promoter usage in tumors with and without *TP53* mutations (Additional file 2: Table S14). Finally, we derived a multivariate Cox proportional hazards model including both *TP53* and *STAG2* mutation status, one candidate gene with differential promoter activity (*CALCB*), and five candidate gene isoforms (*CASZ1*, ENST00000496432; *RET*, ENST00000479913; *TEX40*, ENST00000328404; TNS1, ENST00000446903; and SLC27A6, ENST00000508645), and we followed this with backwards stepwise model selection. The final model (Fig. 5b) revealed potential protective roles for one candidate transcript *TNS1* (ENST00000446903), a marginally protective role for candidate transcript *RET* (ENST00000479913), and a candidate transcript associated with a poor prognosis, *SLC27A6* (ENST00000508645).

*TNS1* encodes the well-studied protein Tensin 1 and is involved in several key aspects of cell function, including extracellular matrix formation, actin cytoskeleton formation, and signal transduction [64, 65]. More recently, the upregulation of TNS1 in colorectal cancer was found to be associated with poor overall survival in patients [66], although previous studies have shown suppression of *TNS1* expression [67] is associated with metastatic cancers. Our results suggest that *TNS1* is a candidate prognostic indicator for EWS. Further studies are needed to draw more attention to the functional roles of these genes/transcripts in EWS progression.

## Discussion

Although epigenetic studies in disease models (cell lines, xenografts, and organoids) and in a limited number of primary tumor samples have demonstrated the oncogenic contributions of epigenetic deregulations to cancer initiation, progression, and response to treatment [48, 68, 69], genome-wide profiling of promoter activities by using standard approaches (e.g., ChIP-seq or CAGE) has not been carried out in large patient tumor cohorts, despite the continuous efforts of large epigenome consortia [45, 46, 70]. Our analyses of *MYCN*-amplified NBL tumors revealed both commonly active promoters and promoters that were active in some tumors but not in others. Moreover, these sample-specific active promoters are functionally important, as they drive the expression of several cancer consensus genes, including *MYC*. This observation is consistent with recent reports of heterogeneous enhancer activities of cell line-defined super-enhancers in primary gastric cancers [71], emphasizing the critical importance of deriving sample-specific epigenomic signatures. To bridge the gap between the extensive epigenomic resources in disease models and the limited ChIP-seq profiles of primary patient tumors, we developed MethylationToActivity (M2A), a deep-learning framework, to characterize the promoter activity landscape (both H3K4me3 and H3K27ac levels) in individual tumors by using DNAm data, which is the most extensively documented epigenetic modification for patient tumors and can be robustly and accurately profiled in FFPE archived retrospective samples. M2A demonstrated excellent performance across various tumor types, with accuracy comparable to that of ChIP-seq measurements of replicate samples from high-quality cohorts (Fig. 3).

MethylationToActivity allows us to answer several questions that have been impossible to address previously. For example, landscapes of comprehensive epigenetic deregulations in promoter regions can be inferred in large primary patient tumor cohorts preserved in FFPE, which previously has been intractable due to technical and financial challenges. The M2A inferred promoter activity landscape will greatly increase sample size, allowing robust statistical analysis to identify recurrent epigenetic deregulations in promoters.

Although our framework was strictly trained on HM levels, the inferred promoter activity was highly correlated with the transcript-based gene expression levels quantified by RNA-seq (Additional file 2: Tables S5, S7 and S8). The correlation between gene expression and inferred promoter activities (mean *R*^2^ = 0.668 for ENCODE data, 0.722 for K562, 0.705 for GM12878, and 0.536 for H1-ESC) surpassed that with the state-of-art BPR model [42], which was developed for predicting gene expression levels from DNAm patterns (the best reported *R*^2^ values were 0.49 for K562, 0.37 for GM12878, and 0.25 for H1-ESC). Strikingly, the correlation of M2A’s predicted promoter H3K27ac activities with gene expression levels across nine ENCODE cell lines (average *R*^2^ = 0.668) was comparable to the predictive accuracy of a model built on 11 HMs, one histone variant, and DNase I hypersensitivity [11]. Although these results are remarkable, we caution that differences in feature design and pre-processing steps (e.g. normalization of HM activity data) may contribute to the apparent high correlation with gene expression levels. Similarly, the accuracy of binary prediction of expressed genes (average AUC = 0.941 and 0.931 for the ENCODE and AML datasets, respectively) surpassed that of DeepChrome (average AUC = 0.80), a state-of-art deep-learning algorithm trained to predict expressed genes by using five core HMs (H3K4me3, H3K4me1, H3K36me3, H3K9me3, and H3K27me3) [12]. These results further validated our framework. Finally, both the M2A and BRP models suggest that it is insufficient to represent DNAm information by using a simple average methylation level in the promoter region. To properly reveal the regulatory roles of DNAm, we need to derive high-order features that capture spatial relations among CpG probes (or window-based derived features calculated from them) in promoters and in their vicinity. M2A uses the feature learning and selection characteristics of CNNs to achieve its exceptional performance, thus demonstrating the rich information content of DNAm signatures at both the genome-wide and local gene levels.

Analysis of the deep-learning framework revealed that M2A derives (1) high-level features from DNAm patterns that are common among different tumors and (2) tumor subtype-specific mapping functions from the mapping of high-level DNAm features to promoter activities in individual tumor subtypes by using transfer learning (when feasible). Although our deep-learning model cannot establish a causal relation between DNAm and promoter activities, these findings nevertheless shed light on both the general and tumor subtype-specific rules for interpreting DNAm patterns.

In evaluating our predictions, we found that several samples (the “poor performers”) showed abnormally low predictive accuracy with both the ENCODE and BLUEPRINT datasets. Investigations of the promoter H3K27ac levels revealed that the fraction of active promoters in these samples was substantially lower than in other samples. Furthermore, joint analyses with RNA-seq data from the matching samples indicated that (1) in contrast to the predictive accuracy of H3K27ac levels, the “poor performers” achieved comparable consistency between the M2A-inferred promoter activity and gene expression; (2) the “poor performers” showed significantly lower correlation between the measured promoter H3K27ac level and gene expression; (3) the correlation between the measured promoter H3K27ac level and gene expression was highly predictive of the accuracy of the H3K27ac prediction (Additional file 1: Fig. S10); and (4) in ENCODE samples with replicates, the replicate with better consistency between H3K27ac and gene expression also showed significantly higher correlation between the actual and measured H3K27ac level (*P =* 0.0039, Wilcoxon signed-rank test, Additional file 2: Table S15, an example of H3K27ac signal discrepancy between H1-ESC replicates shown in Additional file 1: Fig. S8d). Although we cannot unequivocally rule out the possibility that these “poor performers” share a distinct biological mechanism where promoter H3K27ac level is no longer a stronger predictor for gene activities, these results suggested that the “poor performers” could reflect the quality of the ChIP-seq results included in the test data. This observation emphasizes the value of conducting a preliminary analysis to evaluate the data quality before incorporating public data. It also suggests that M2A can provide a robust surrogate for promoter activities when the ChIP-seq experimental data is questionable.

Alternative promoter usage increases the transcriptomic diversity during normal tissue development and oncogenesis. Recent work demonstrated that an alternative promoter of *ERBB2* is predictive of a poor clinical outcome but that the canonical promoter shows no significant association with survival in patients with low-grade glioma [2]. Whereas earlier studies focused on identifying alternative promoter usage through CAGE or RNA-seq data, our research has shown that alternative promoter usage can be extensively studied by using DNAm profiles from diverse samples, including retrospective FFPE tumor samples, for which traditional approaches (CAGE, ChIP-seq, and RNA-seq) are technically challenging. Our analysis of a large EWS cohort (without matching RNA-seq data) revealed promoter activities for several specific isoforms that are independently associated with clinical outcomes, including a specific isoform of the *TNS1* gene (ENST00000446903). This demonstrates the importance of analyzing alternative promoter usage in epigenomic studies.

Finally, our analysis quantitatively emphasizes that promoter activity is one of the mechanisms that regulate the final transcriptional output: (1) both the observed and predicted promoter activities account for 50–70% of the variation in gene expression, and (2) approximately 40% of the genes differentially expressed in ERMS and ARMS tumors show significant differences in their promoter activities. In addition to promoter activities, other epigenetic mechanisms, including enhancer activities, contribute substantially to gene regulation [72]. Recent work has demonstrated the roles of DNAm in regulating enhancer activities [73] and aberrant cancer-specific DNAm patterns in super-enhancers [74]. Consequently, we aim to expand our M2A framework to infer enhancer activities from DNAm patterns in the future.

## Conclusion

We have demonstrated that MethylationToActivity overcomes the unique challenges of systematically characterizing promoter activities from DNA methylation signatures. It achieved an accurate, robust, and generalizable performance in various pediatric and adult cancers, including both solid and hematologic malignant neoplasms. MethylationToActivity will serve as a valuable tool to provide functional interpretation of DNAm deregulation, characterize promoter activity differences from DNAm patterns, and reveal alternate promoter usage in patient tumors, which will facilitate precision medicine by tailoring treatments based on both genetic variants and epigenetic deregulation.

## Methods

### Datasets

Five separate publicly available datasets were used in this study, including a pediatric NBL O-PDX dataset (*N* = 16) [43]; an RMS O-PDX dataset (N = 16) [44]; ENCODE datasets with matching H3K27ac and H3K4me3 histone mark ChIP-seq, RNA-seq, and WGBS experimental data (*N* = 9) [45]; a DCC BLUEPRINT AML dataset (*N* = 19) [46]; and a pediatric EWS dataset (*N* = 140) [22] (Additional file 2: Table S16). Of the 140 samples in the EWS cohort, only three had matching ChIP-seq and reduced-representation bisulfite sequencing (RRBS) data available; for the remaining 137 samples, only RRBS data was available. All other cohort datasets (i.e., the RMS, NBL, ENCODE, and AML datasets) contained matching H3K27ac and H3K4me3 profiles, along with RNA-seq and WGBS experimental data.

### Feature processing

All datasets were evaluated using GENCODE annotation definitions (www.gencodegenes.org/); the NBL, RMS, ENCODE, and EWS datasets were evaluated using GENECODE GRCh37.p13 (release 19), and the AML dataset was evaluated using GENCODE GRCh38.p13 (release 32). Promoter regions are defined as the TSS ± 1 kbp, where the TSS is defined as each unique transcript start position. To avoid using identical or near-identical promoter regions in training and baseline performance, only TSSs with promoter regions with less than 50% overlap were considered. Gene orientation was taken into account, and any promoters with overlying regions but opposite orientations were not considered as overlapping. Because of differences in sex among samples, all chromosome X and Y promoter regions were removed from consideration. This resulted in a total of 96,756 and 104,722 non-overlapping promoter regions from the annotation files GRCh37.p13 and GRCh38.p13, respectively. For gene expression analysis, we followed the definition in [11] and retained all 141,152 and 147,980 autosomal promoters for protein-coding genes from the annotation files GRCh37.p13 and GRCh38.p13, respectively.

M2A uses only one variable feature type: DNA methylation. For WGBS/RRBS data, the *M*-value was calculated as follows:

$$ {Mval}_k={\log}_2\left(\frac{{\mathrm{Methylated}}_k+\alpha }{{\mathrm{Unmethylated}}_k+\alpha}\right), $$

where Methylated_*k*_ and Unmethylated_*k*_ correspond to the number of methylated and unmethylated reads of the *k*th CpG site, respectively. By default, the offset *α* was set to 0.5, and a global *M*-value threshold was set to a maximum value of log_2_(65) and a minimum value of ${\log}_2\left(\frac{1}{65}\right)$. CpG sites with coverage of less than five reads were removed.

M2A uses a promoter region-based windowed approach, comprising 20 windows of two sizes (250 bp and 2.5 kbp) and a step size equal to the window size, centered on a given TSS. For instance, ***W***_*ij*_ = {*W*_*i*1_, *W*_*i*2_, *W*_*i*3_…, *W*_*i*20_} is the vector of windows where ***i*** represents a particular TSS and ***j*** represents a particular window corresponding with the *i*th TSS. This means that *W*_*i*10_ and *W*_*i*11_ represent the windows immediately downstream and upstream of the *i*th TSS. Therefore,

$$ {W}_{i1}={\mathrm{TSS}}_i-{10}^{\mathrm{th}}\ \mathrm{window}, $$

*W*_*i*2_ = TSS_*i*_ − 9^th^ window, **…**

$$ {W}_{i19}={\mathrm{TSS}}_i+{9}^{\mathrm{th}}\ \mathrm{window}, $$

$$ {W}_{i20}={\mathrm{TSS}}_i+{10}^{\mathrm{th}}\ \mathrm{window} $$

Each feature was calculated in this manner. The DNA methylation features, including the windowed *M*-value mean, variance, and the fraction of the SSD of *M*-values (FSSD), were calculated and represented by the feature vectors *M*ave_*i*_, *M*var_*i*_, and *M*fssd_*i*_. Therefore, the features for a particular window, denoted as *W*_*ij*_, would be calculated as follows:

$$ {M\mathrm{ave}}_{ij}=\frac{1}{n_{ij}}\sum {M\mathrm{val}}_{ik(j)}, $$

$$ M{\operatorname{var}}_{ij}=\frac{1}{n_{ij}}\sum {\left({M\mathrm{ave}}_{ij}-{M\mathrm{val}}_{ik(j)}\right)}^2, $$

and

$$ {Mf\mathrm{ssd}}_{ij}=\frac{{M\mathrm{ssd}}_{ij}}{\sum {\left({M\mathrm{ave}}_i-{M\mathrm{val}}_k\right)}^2}, $$

where

$$ {M\mathrm{ave}}_i=\frac{1}{n_i}\sum {M\mathrm{val}}_{k.} $$

and

$$ {M\mathrm{ssd}}_{ij}=\sum {\left({M\mathrm{ave}}_{ij}-{M\mathrm{val}}_{ik(j)}\right)}^2, $$

Here, *i* represents the promoter, *j* represents a specific window for a particular promoter, and *M*val_*k*_ represents the *M*val for individual CpGs in a region where *M*val_*k*(*j*)_ is the Mval for an individual CpG in a specific window. Each feature was interleaved by window size to provide model input wherein each window contained a number of “channels” equal to the number of features, with the feature array shape being (*N*, 2, 20, 3), where *N* represents the total number of TSSs in a sample, 2 represents the number of window sizes (250 bp and 2.5 kbp), 20 represents the number of windows, and 4 represents the number of features per window. All features were scaled from 0.1 to 1 (using MinMaxScaler with default values from sklearn version 0.22); in instances where windows overlapped regions without methylation data, resulting in NaNs (such as chromosomal boundaries, telomeric regions, and centromeric regions), these feature values were marked as 0.

### Calculating histone modification enrichment

The response variable was calculated for each non-overlapping promoter region, a 2000-bp region centered on each TSS. Histone modification enrichment (HM) for the *i*th promoter region is calculated as follows:

$$ {\mathrm{HM}}_i={\log}_2\frac{\sum_k{\mathrm{ChIP}}_{ik}+\alpha }{\sum_k{\mathrm{Input}}_{ik}+\alpha }, $$

where ${\sum}_k{\mathrm{ChIP}}_{ik}$ represents the sum of either the H3K27ac or the H3K4me3 read signal mapped to the promoter region at each position, ${\sum}_k{\mathrm{Input}}_{ik}$ represents the sum of the control read signal mapped to the promoter region, and *α* represents the 25th percentile of the ${\sum}_k{\mathrm{Input}}_{ik}$ calculated for a given sample.

### M2A topology

M2A is a machine learning framework that leverages canonical deep-learning strategies, including convolutional neural network (CNN) and fully connected (FC) layers. Each layer employs a LeakyReLU (alpha = 0.1) and a kernel constraint by L2-normalization using maxnorm (3). CNN layers are two-dimensional, with zero padding, a stepsize of (1,1), and a kernel size of (1, 3) to maintain feature space and prevent convolutions across features from different window sizes. To test the efficacy of this approach, we compared the performance of a traditional artificial neural network (ANN) consisting of two FC layers versus three CNN layers in addition to two FC layers. During transfer learning, weights corresponding to each of the three CNN layers of the six-NBL O-PDX M2A model trained previously were frozen; only the weights corresponding to the two FC layers were optimized. A summary of all model topologies and parameters can be found in Additional file 2: Table S17. To train and test each model topology, we used Keras (v2.2.4) and Tensorflow (v2.1.0) in Python 3.6.5.

### M2A parameter tuning

Parameters such as the window size, batch size, and kernel size were optimized using the validation NBL data set (*n* = 10). Each parameter configuration was tested holding all other parameters constant, and models with a numerical performance advantage were chosen. For batch size, three configurations were tested (64, 128, 256). Four kernel size configurations ([1–5]) were tested, and window configurations ([100 bp, 1000 bp], [250 bp, 2500 bp], [500 bp, 5000 bp]) were considered. Due to > 50% uninformative features in the 100-bp window resolution, only the (250 bp, 2500 bp) model and (500 bp, 5000 bp) model performances were compared (Additional file 1: Fig. S13; Additional file 2: Table S18).

### Training M2A

The core M2A model (without transfer) training set consisted of six O-PDX samples from the 16-sample NBL cohort (Additional file 2: Table S19); we trained separate models for H3K27ac and H3K4me3 HMs with the same WGBS features as the input. After the base models were trained, transfer learning was employed for three separate datasets, namely the RMS, AML, and EWS datasets. Each transfer learning model was trained using one sample from the cohort, for a total of *N* models, where *N* equals the number of samples in the cohort. For the RMS and EWS cohorts, an ensemble approach was used, whereby an averaged prediction from *N* − 1 models was generated after transfer learning with each sample. The same approach was used with the AML cohort, except that only samples with *R*^2^ ≥ 0.60 between FPKM and H3K27ac were used for transfer learning.

For each training scheme, the same parameters were used, including an 80/20 training/validation split and a batch size of 64 (Additional file 2: Table S17). All sample input was randomized before training. The Keras implementation of adadelta (default parameters) minimizing the mean squared error (MSE) was used to optimize M2A. To prevent overtraining, the EarlyStopping method was employed by monitoring validation loss for 10 epochs without at least a minimal gain in performance (min_delta = 0.0001) for a maximum of 80 epochs. In no case was the maximum number of epochs reached.

### Determining promoter diversity in NBL models

Promoter region-based H3K27ac distributions clearly show a bimodal distribution (see Additional file 1: Fig. S1); therefore, to determine class occupancy (active versus inactive), a Gaussian mixture model (GaussianMixture from sklearn version 0.22 with n_components = 2) was applied for each individual sample. To determine the percentage of differentially active promoters among all active promoters, we used a pairwise comparison approach for all samples. Cancer consensus genes were downloaded from COSMIC (https://cancer.sanger.ac.uk/census) (accessed on February 1, 2020). To avoid artificially inflated values from genes with multiple TSSs, only cancer consensus genes with a single TSS according to GENCODE GRCh37.p13 (release 19) definitions were considered.

### Evaluating M2A performance

When determining prediction performance, two primary metrics were considered, namely the *R*^2^ and the root mean squared error (RMSE). To measure the accuracy of M2A in predicting expressed genes, we calculated the AUC-ROC by using roc_curve and the precision-recall curve AUC by using average_precision_score from sklearn 0.22. Paired analyses were tested for statistical significance by using a Wilcoxon signed-rank test (R v3.4.1). To determine outliers, a median-based method was implemented using the “outlier” function in the R package GmAMisc [55]. To ensure that low-mappability regions were not a confounding factor, we used the wgEncodeCrgMApabilityAlign100mer.bw file downloaded from the UCSC Genome Browser (http://genome.ucsc.edu/cgi-bin/hgFileUi?db=hg19&g=wgEncodeMapability). For GRCh38.p13 annotations, we used liftOver from UCSC (http://hgdownload.cse.ucsc.edu/admin/exe/linux.x86_64/) to convert the mappability track to GRCh38. For all performance-related analyses, the average value of this track within each non-overlapping 2000-bp promoter region was calculated, and promoter regions with mappability > 0.75 were retained for evaluation.

In analyses between H3K27ac levels (actual or predicted) and gene expression, we followed the gene filtering steps described in [11]: all autosomal protein-coding gene promoters were considered and for genes with multiple promoters, we use the maximum promoter activity to represent the gene.

### Baseline models

Multivariate adaptive regression splines (MARS) was implemented by “earth” package in R (all default values were used), and the random forest baseline model was implemented by the “sklearn.ensemble. RandomForestRegressor” package in python 3.7.0 (max_features = ‘sqrt’). For comparison purposes, each model tested used identical feature input to M2A.

### M2A captures the impact of DMRs on promoter region activity

The lists of genes that are differentially expressed in ERMS and ARMS samples and genes with DMRs were previously reported in reference [42].

### Alternative promoter usage analyses

To infer alternative promoter usage that was specific to the RMS subtypes ARMS and ERMS, we first delineated the “active” vs. “inactive” promoters in a subtype (ERMS or ARMS) by applying a threshold of mean(*H*3*K*27*ac*) > 1 for the average samples in the subtype. Next, primary promoters from multi-promoter genes were determined by the average H3K27ac level within a specific subtype, and the promoter with the maximum activity from a given gene was counted as the primary promoter. The differences in promoter usage between two subtypes were defined as the difference between the activity sum of the primary promoters and the activity sum of the secondary promoters in the two subtypes.

### Analysis of alternative promoter usage in EWS

In the same manner as the RMS subtype analysis, EWS alternative promoter usage was determined in two patient sample groups: *TP53* mutant and *TP53* wild-type groups (for sample status, see Additional file 2: Table S18). Candidate genes were identified as active genes (H3K27ac ≥ 1 in at least one group) with potential differential promoter activities (absolute difference ≥ 1) in the *TP53* wild-type and *TP53* mutant groups. Candidate genes with alternative promoter usage were identified as genes that used different active primary promoters in the wild-type and mutant groups and had an average promoter usage difference of at least 0.4 between groups. Both alternative promoters and differentially active promoters were considered in prognostic analyses (univariate screening incorporating both *TP53* and *STAG2* mutation status, followed by a multivariate analysis) using Cox proportional hazard models (R 3.4.1). The final model was derived from backward stepwise selection from a Cox proportional hazards model including *TP53* and *STAG2* mutation status and all potential markers (all genes or promoters with an FDR < 0.05 in the univariate analysis).

### Analysis of M2A feature input and extracted features

To determine the merit of a CNN-based approach, each feature average for a particular window position and window size were plotted with a 95% confidence interval. The plotted feature distribution was calculated from all M2A vanilla model training data input (NBL, *N* = 6) and stratified by promoter status. Promoter status was determined by class occupancy of both H3K27ac and H3K4me3 (active versus inactive), where (H3K27ac = active), (H3K27ac = inactive, H3K4me3 = active), and (H3K27ac = inactive, H3K4me3 = inactive), represent active, poised, and inactive promoters, respectively. Class occupancy was determined by applying a Gaussian mixture model (GaussianMixture from sklearn version 0.22 with n_components = 2).

The efficacy of the CNN-based feature extraction was tested by (1) training sample input feature predictive performance as compared to CNN-extracted feature performance, calculated by Pearson’s *R*^*2*^ for all features across the entire training set (each window size considered separately; NBL, *N* = 6) and (2) the performance (Pearson’s *R*^*2*^) of the best performing feature identified in (1) when applied to each sample in the validation set (NBL, *N* = 10; Additional File 1: Fig. S3).

### Availability of data and materials

The datasets used in this report have been published, including a pediatric NBL O-PDX dataset (*N* = 16) [43, 75]; an RMS O-PDX dataset (*N* = 16) [44, 76]; ENCODE datasets (downloaded from https://www.encodeproject.org/) with matching H3K27ac and H3K4me3 histone mark ChIP-seq, RNA-seq, and WGBS experimental data (*N* = 9) [45, 77]; a DCC BLUEPRINT AML dataset (*N* = 19) [46, 78]; and a pediatric EWS dataset (*N* = 140) [22, 79, 80]. All relevant accession numbers can be found in Additional file 2: Table S16 and Table S19.

The latest M2A models, feature generation, prediction pipeline, and a Docker image of the M2A environment pre-loaded are available for download at https://github.com/chenlab-sj/M2A, and the latest release is hosted by Zenodo [81], under the Apache License 2.0. Additionally, source code with detailed instructions for transfer learning using the M2A model with input samples from other domains is available. The cloud-based implementation of M2A is available to anyone with a (free) St. Jude Cloud account (https://platform.stjude.cloud/workflows/methylation-to-activity).

## Supplementary Information

**Additional file 1: Figure S1.** NBL sample H3K27ac promoter distribution. **Figure S2.** DNAm input feature pattern analysis. **Figure S3.** Feature performance comparison: Input features vs CNN mapped features. **Figure S4.** M2A prediction generalizability analysis. **Figure S5.** M2A with transfer learning outperforms a vanilla M2A model of the same cancer type. **Figure S6.** Signal-to-noise analysis of ENCODE and NBL datasets. **Figure S7.** M2A ENCODE cohort performance. **Figure S8.** Analysis of outlier H1-ESC. **Figure S9.** Predicting gene expression in the ENCODE dataset. **Figure S10.** Consistency of gene expression and H3K27ac promoter levels in the AML cohort. **Figure S11.** M2A accurately determines subtype differences between embryonal and alveolar RMS. **Figure S12.** Kaplan–Meier log-rank analysis by mutation status in EWS. **Figure S13.** CpG distribution by window relative to the TSS.

**Additional file 2: Table S1.** H3K27ac active cancer consensus genes in 3 NBL cell lines, and 3 NBL O–PDX samples. **Table S2.** Baseline models vs. vanilla M2A predictive performance comparison. **Table S3.** M2A predictive performance in NBL cell line samples. **Table S4.** Observed H3K27ac and H3K4me3 ENCODE replicate consistencies. **Table S5.** M2A predictive performance in RMS O–PDX samples. **Table S6.** M2A RMS transfer model predictive performance in RMS O–PDX samples. **Table S7.** M2A predictive performance in ENCODE dataset. **Table S8.** M2A predictive performance in AML samples. **Table S9.** ERMS vs. ARMS DMRs and associated genes (overexpressed in ERMS). **Table S10.** ERMS vs. ARMS DMRs and associated genes (overexpressed in ARMS). **Table S11.** M2A alternate promoter usage predictive performance between ARMS and ERMS samples. **Table S12.** M2A predictive performance in EWS samples, before and after transfer. **Table S13.** A univariate survival analysis of differential H3K27ac promoter activity between TP53 mutant and TP53 wild–type EWS tumors. **Table S14.** A univariate survival analysis of alternate promoter usage between TP53 mutant and TP53 wild–type EWS tumors. **Table S15.** ENCODE H3K27ac replicate consistency with gene expression. **Table S16.** Dataset availability. **Table S17.** M2A model topologies. **Table S18.** Parameter tuning: mean performance in the NBL validation set (*R*^*2*^). **Table S19.** Sample summary information.

**Additional file 3.** Review history.
