# Supplementary material for: MethylationToActivity: a deep-learning framework that reveals promoter activity landscapes from DNA methylomes in individual tumors
Source: Genome Biol. 2021 Jan 19;22:24. doi: 10.1186/s13059-020-02220-y (PMC7814737; doi:10.1186/s13059-020-02220-y)
Supplement: Supplementary file 1 — Additional file 1: Figure S1. NBL sample H3K27ac promoter distribution. Figure S2. DNAm input feature pattern analysis. Figure S3. Feature performance comparison: Input features vs CNN mapped features. Figure S4. M2A prediction generalizability analysis. Figure S5. M2A with transfer learning outperforms a vanilla M2A model of the same cancer type. Figure S6. Signal-to-noise analysis of ENCODE and NBL datasets. Figure S7. M2A ENCODE cohort performance. Figure S8. Analysis of outlier H1-ESC. Figure S9. Predicting gene expression in the ENCODE dataset. Figure S10. Consistency of gene expression and H3K27ac promoter levels in the AML cohort. Figure S11. M2A accurately determines subtype differences between embryonal and alveolar RMS. Figure S12. Kaplan–Meier log-rank analysis by mutation status in EWS. Figure S13. CpG distribution by window relative to the TSS. [file 13059_2020_2220_MOESM1_ESM.pdf]

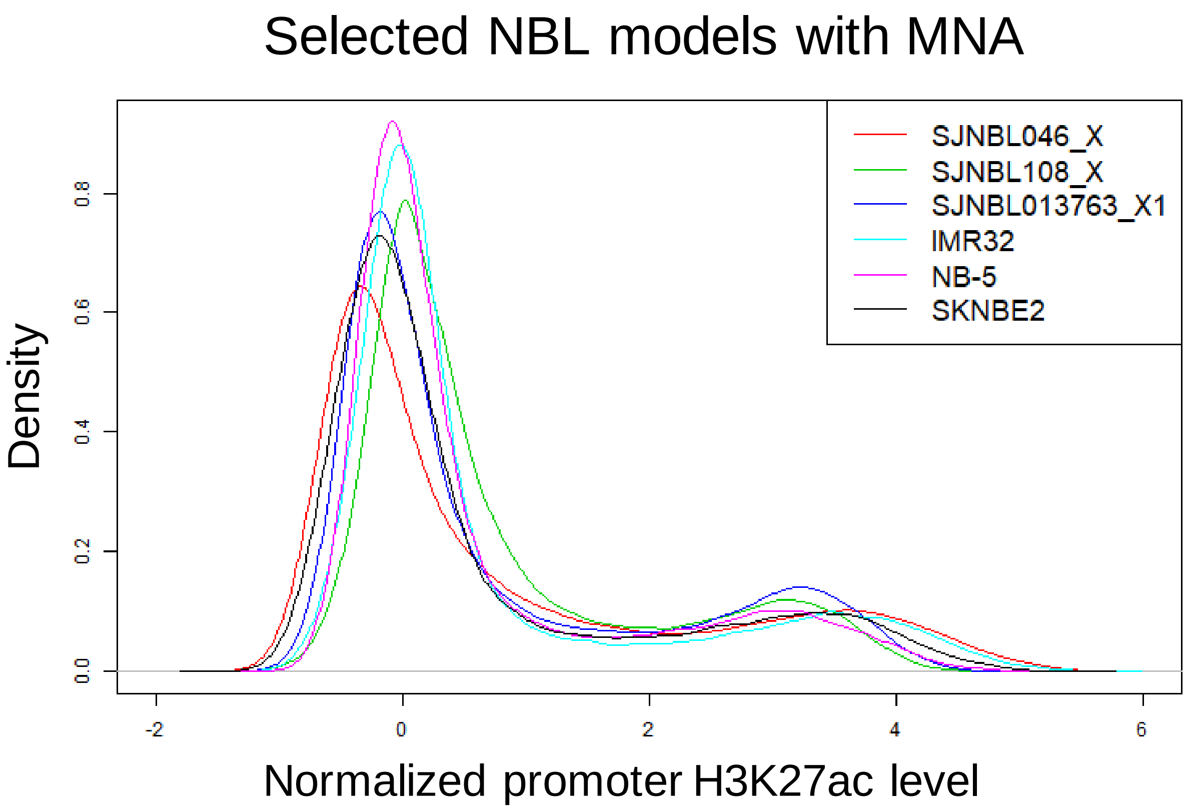

Figure S1. NBL sample H3K27ac promoter distribution.

A comparison of promoter H3K27ac enrichment in *MYCN* amplified (MNA) NBL cell line and O-PDX samples shows a clear bimodal distribution, delineating “active” and “non-active” promoters.

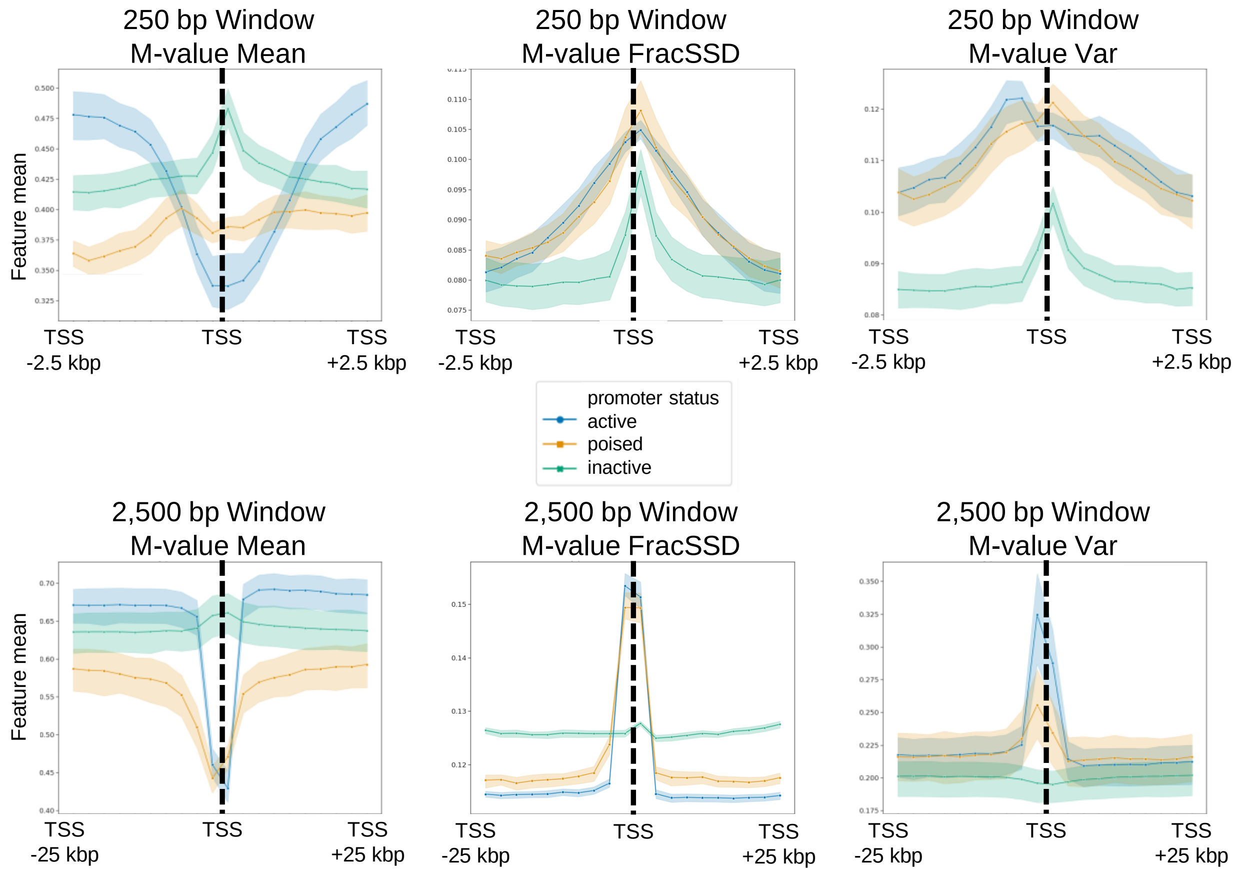

Figure S2. DNAm input feature pattern analysis.

The DNAm features show promoter-status-specific patterns between active, poised, and inactive promoters, emphasizing the utility of positional-based relationships in a windowed DNAm feature approach. For both 250 bp and 2,500 bp window sizes, the average scaled input feature was plotted in relationship to the TSS (ribbons represent the 95% confidence interval), stratified by the promoter status. Promoter status was determined by class occupancy of both H3K27ac and H3K4me3, either “active” or “inactive”, where (H3K27ac=active), (H3K27ac=inactive, H3K4me3=active), and (H3K27ac=inactive, H3K4me3=inactive), represents active, poised, and inactive promoters, respectively.

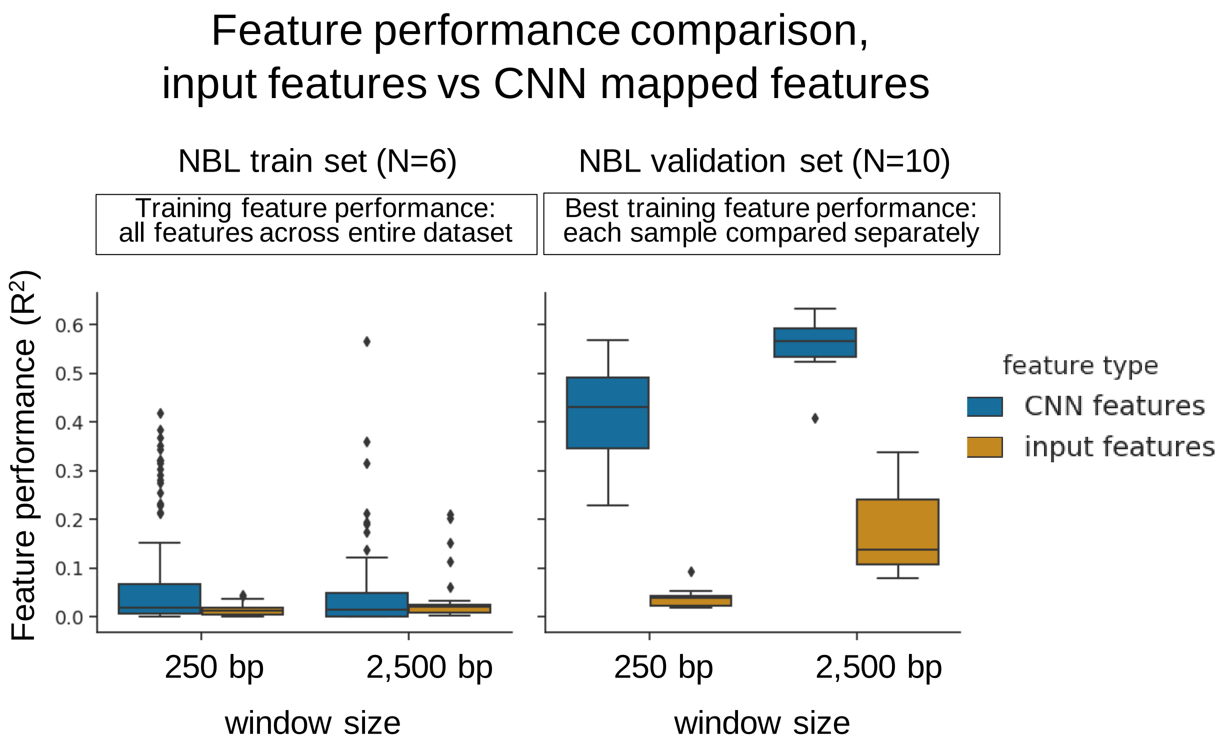

Figure S3. Feature performance comparison: Input features vs CNN mapped features. Using all samples included in training the vanilla M2A model (NBL, N=6), the individual feature performance (as determined by Pearson's R^2 between the feature and the response variable, H3K27ac) for each feature was plotted, comparing the distribution of performances between raw input training feature and the CNN mapped features at a particular window size (250 bp or 2,500 bp). The best feature from this analysis for each window size and feature type (input or CNN mapped) was used to determine Pearson's R^2 with H3K27ac from each sample in the NBL validation set (N=10).

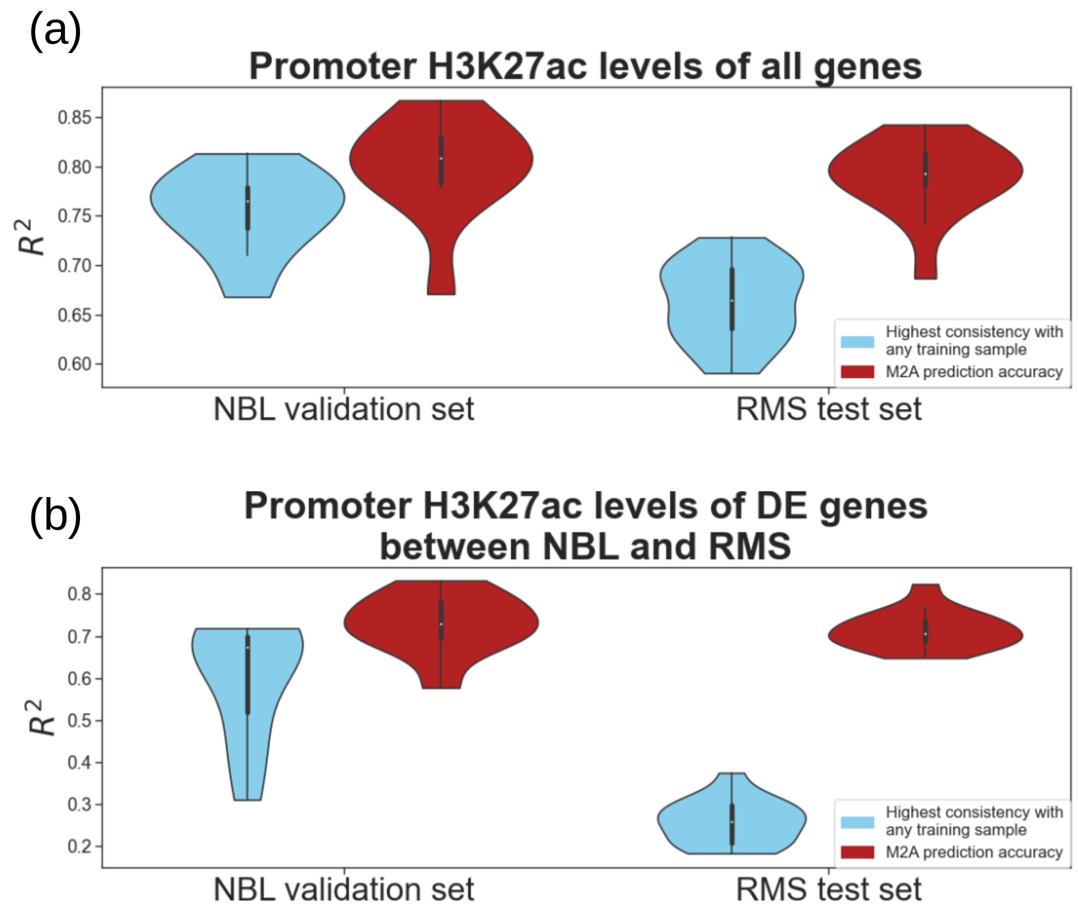

Figure S4. M2A prediction generalizability analysis.

(a) A comparison between performance of the M2A model (R^2 of observed H3K27ac promoter levels and predicted levels in the test sample) with the surrogate model (represented by the highest R^2 of observed H3K27ac promoter levels in the test sample and the observed H3K27ac promoter levels in any training sample). M2A extracts generalizable features capable of out-performing the surrogate model in both the NBL validation set and the RMS test set, further highlighted in **(b),** a comparison of surrogate model and M2A model performance using promoters from DE genes between NBL and RMS.

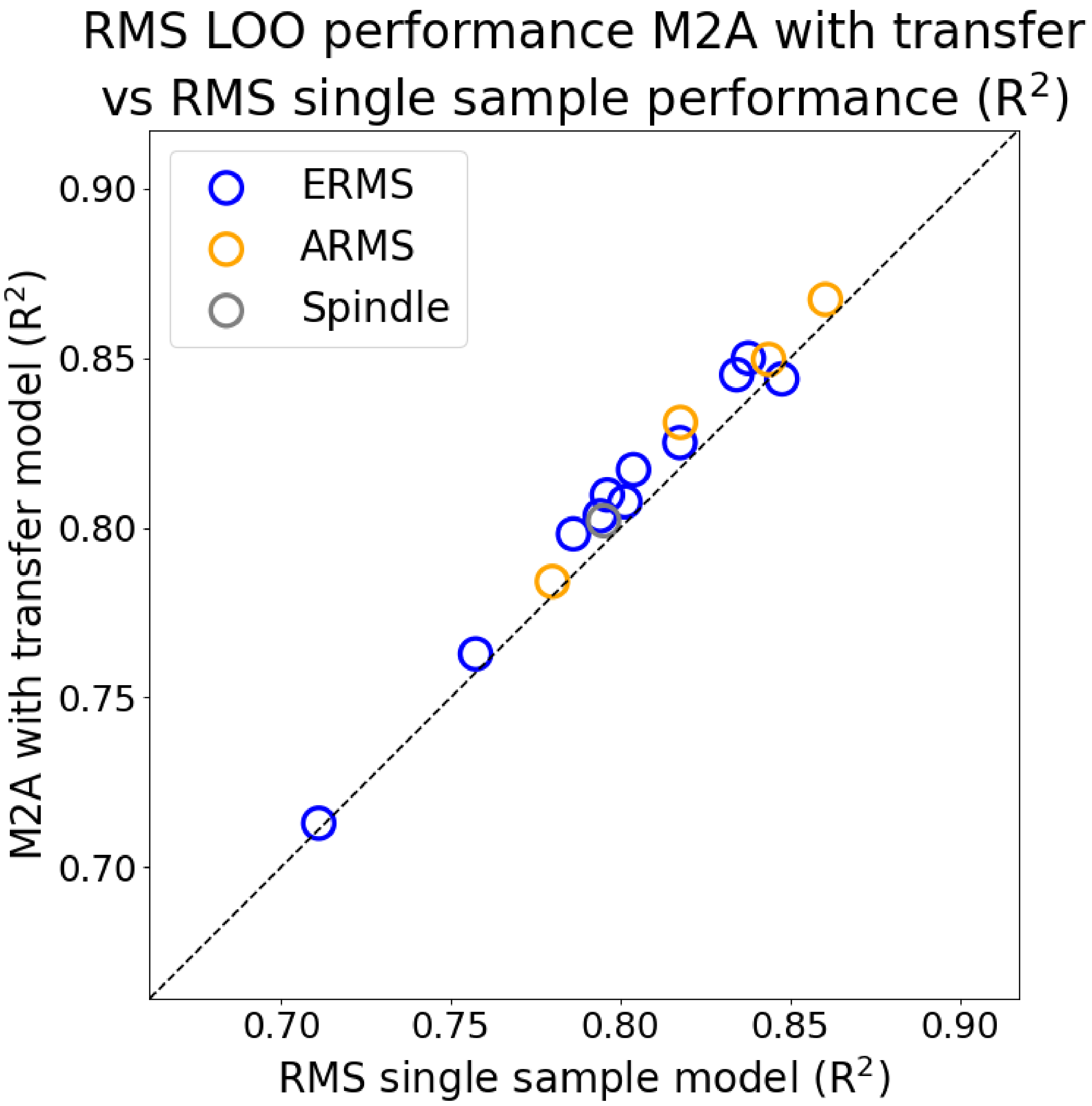

Figure S5. M2A with transfer learning outperforms a vanilla M2A model of the same cancer type.

An M2A model with transfer learning (initially trained with six NBL O-PDX samples and then transferred with a single RMS sample) consistently outperforms an M2A model trained with a single RMS sample.

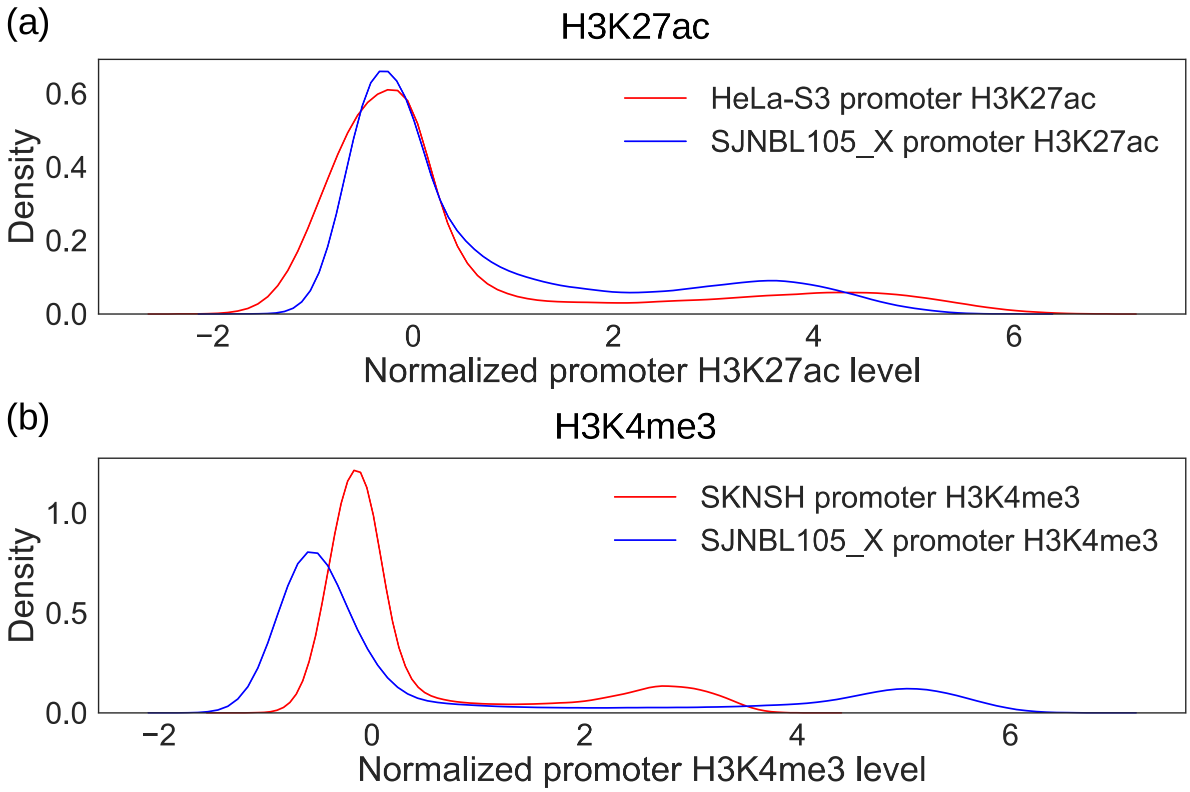

Figure S6. Signal-to-noise analysis of ENCODE and NBL datasets.

Comparison at the observed H3K27ac promoter level **(a)** and the H3K4me3 **(b)** promoter levels revealed different signal-to-noise profiles between the ENCODE dataset and the NBL datasets, which results in a highly correlated prediction with larger RMSE values.

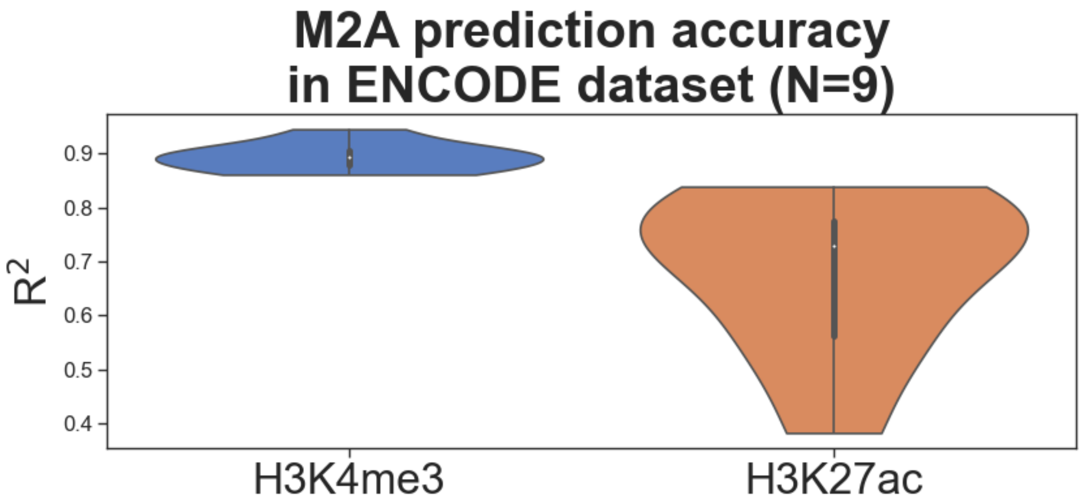

Figure S7. M2A ENCODE cohort performance.

The distribution of M2A prediction performance (R^2), shows that M2A accurately infers both H3K27ac and H4K4me3 promoter levels in the publicly available ENCODE dataset.

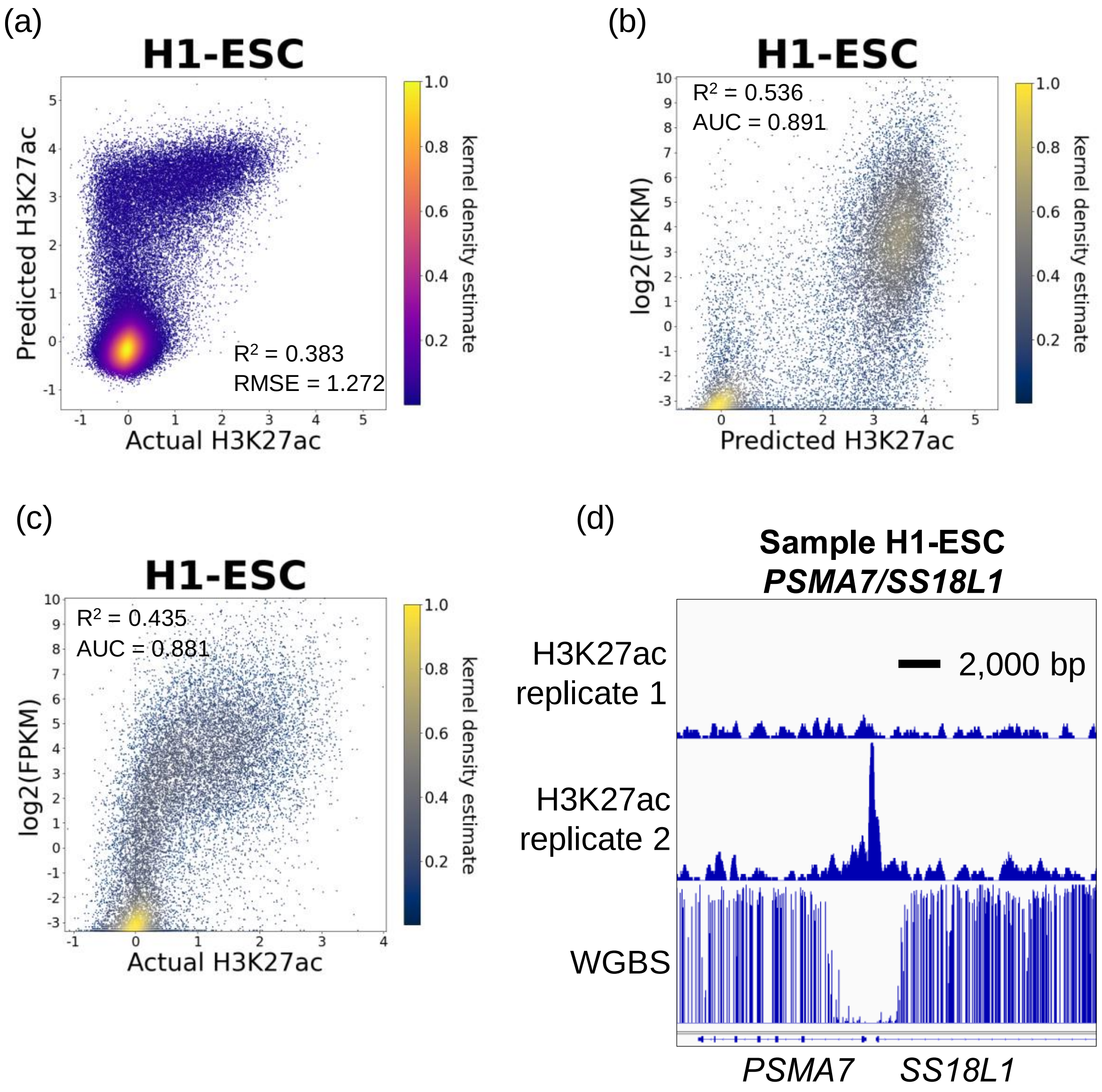

Figure S8. Analysis of outlier H1-ESC.

(a) When inferring H3K27ac promoter levels, M2A was substantially less accurate in the ENCODE sample H1-ESC, which is an outlier in the ENCODE cohort. **(b, c)** M2A-predicted H3K27ac promoter levels **(b)** are more consistent with, and more predictive of, H1-ESC gene expression than are the actual observed H1-ESC H3K27ac promoter levels **(c)**. (**d**) The hypomethylated region surrounding the promoters of genes *PSMA7* and *SS18L1* (often

indicative of H3K27ac enrichment) showed inconsistent H3K27ac levels between H1-ESC ChIP-seq replicates from ENCODE.

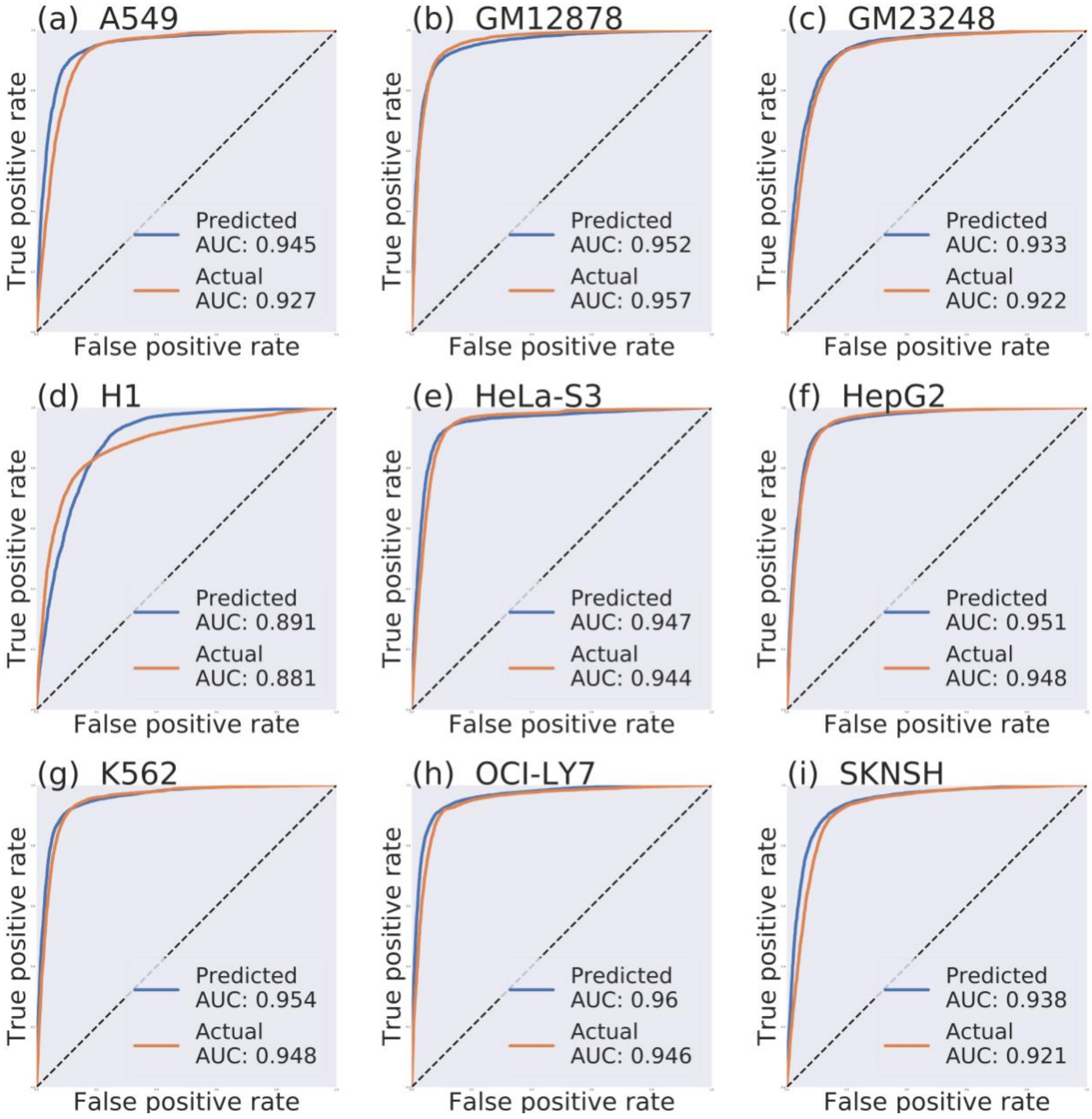

Figure S9. Predicting gene expression in the ENCODE dataset.

(a–i) The indirect ability of M2A to predict gene expression (i.e., expressed vs. not expressed) on the basis of both M2A-predicted and observed H3K27ac promoter levels for each sample was determined by comparing the AUCs of the receiver operating characteristic (ROC) curves.

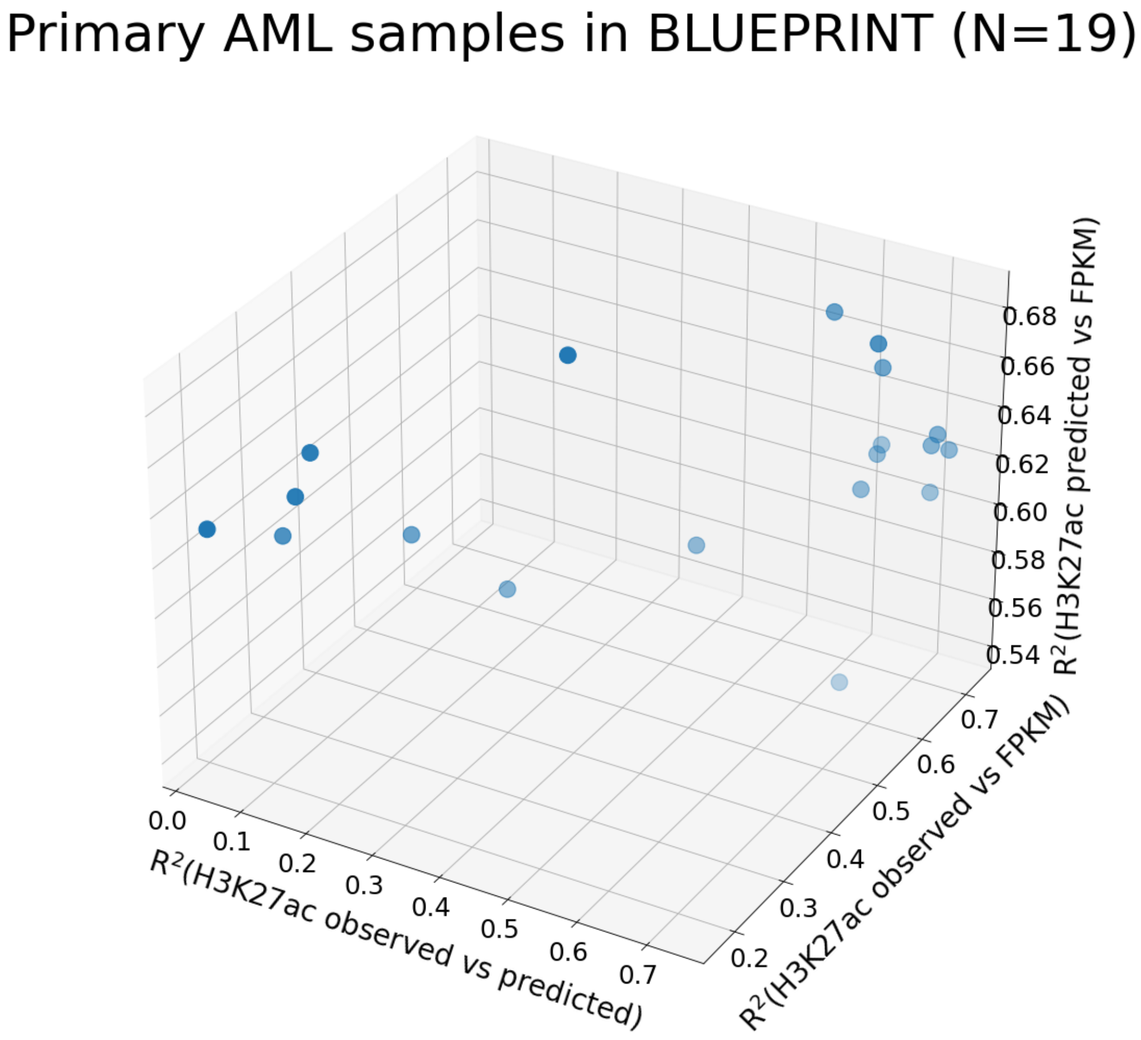

Figure S10. Consistency of gene expression and H3K27ac promoter levels in the AML cohort.

The consistency, as determined by Pearson's R^2, between the observed values for gene expression and the H3K27ac promoter levels is remarkably predictive of the performance M2A in predicting H3K27ac promoter levels in samples from the AML cohort.

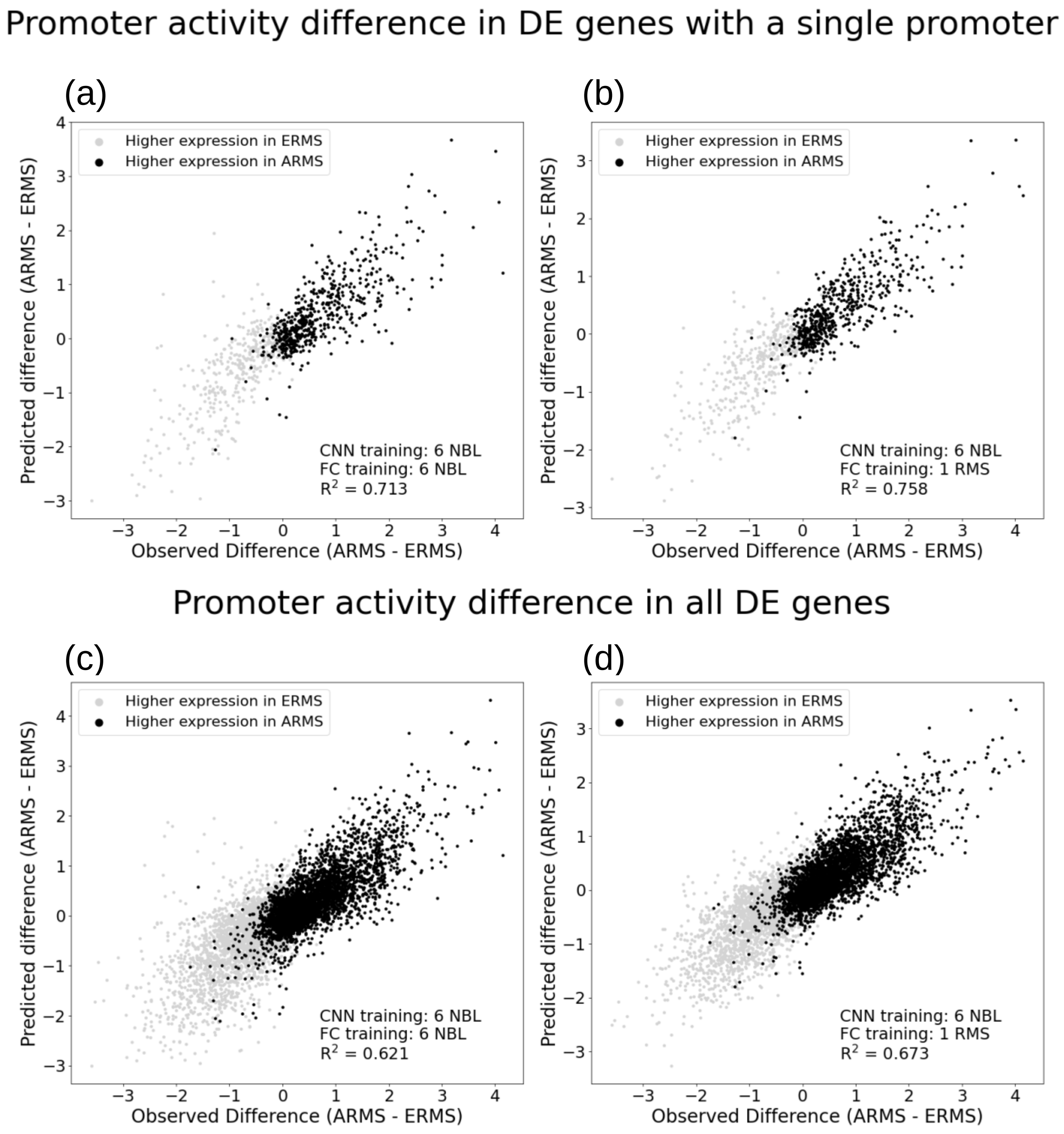

Figure S11. M2A accurately determines subtype differences between embryonal and alveolar RMS.

(a) The promoter activities of single-promoter, differentially expressed genes in the RMS subtypes ERMS and ARMS are accurately inferred by an M2A base model (trained with six O-PDX NBL samples). **(b)** The predictive performance of M2A is further boosted by transfer

learning with one RMS sample. **(c, d)** The performance of M2A declines slightly when the model is applied to all promoters of differentially expressed genes **(c)**, but it recovers when an M2A model with transfer learning with only one RMS training sample is applied **(d)**.

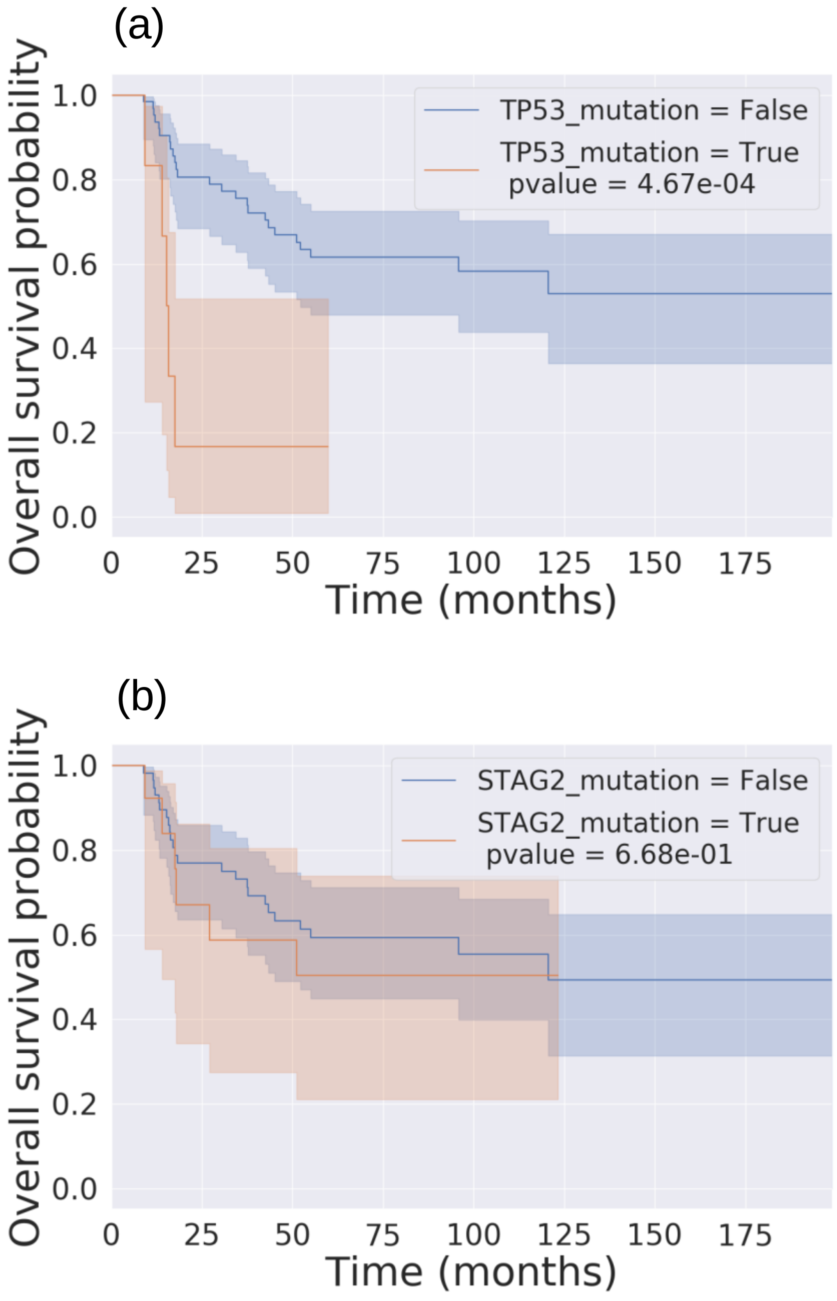

Figure S12. Kaplan–Meier log-rank analysis by mutation status in EWS.

The prognostic ability of **(a)** *TP53* or **(b)** *STAG2* mutation status in the EWS cohort was determined by the log-rank test and visualized using the Kaplan–Meier survival curve. The *STAG2* mutation status showed no significant difference in overall survivability, thus only *TP53* mutation status was considered when forming the univariate and multivariate Cox proportional hazards model.

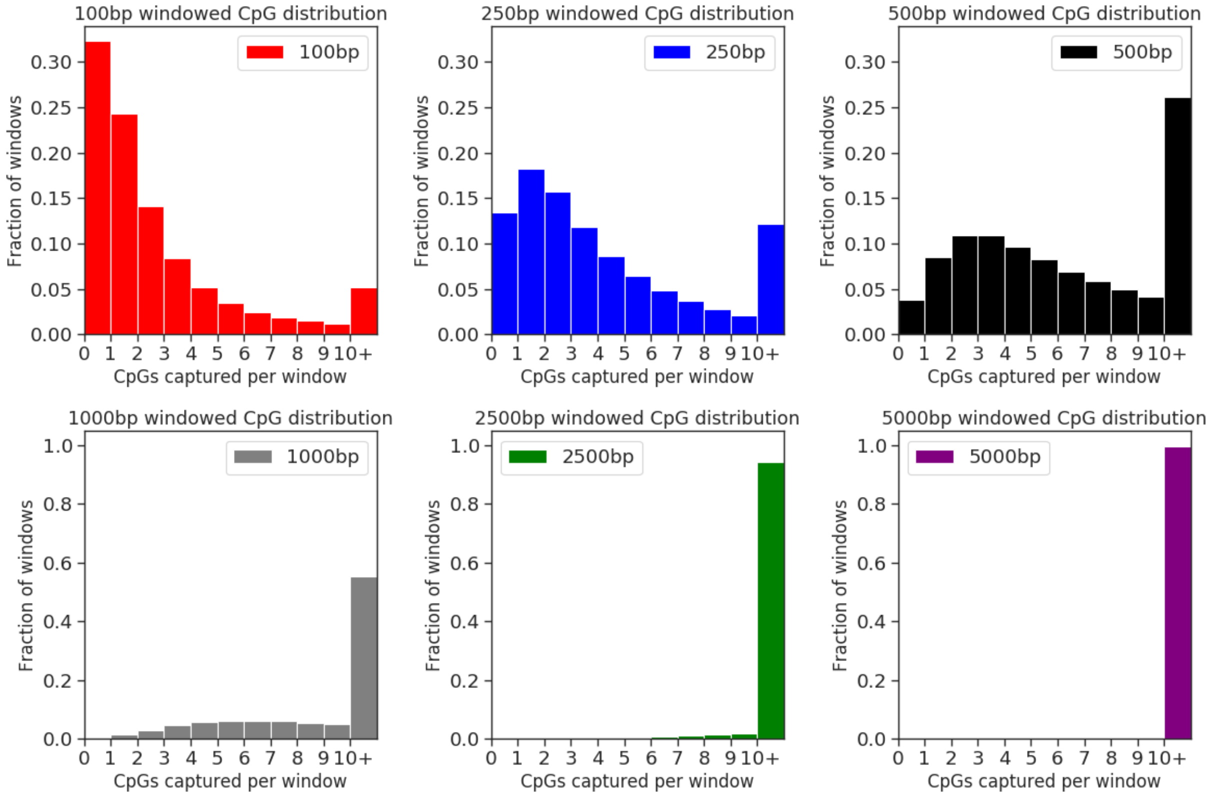

Figure S13. CpG distribution by window relative to the TSS.

To achieve feature input that is informative to the model, M2A window size selection was partially based on the number of CpGs captured by window size. Each analysis consists of 20 windows surrounding each TSS at a particular window size, representing the theoretical CpG input to M2A for that particular resolution. Three different window configurations were considered, comprised of two window sizes: 1) 100 bp and 1,000 bp, 2) 250 bp and 2,500 bp,

and 3) 500 bp and 5,000 bp. Due to NaNs in feature windows calculated with fewer than 2 CpGs, the [100 bp, 1000 bp] model was removed from consideration (> 50% NaNs).
