# Supplementary material for: MethylationToActivity: a deep-learning framework that reveals promoter activity landscapes from DNA methylomes in individual tumors
Source: Genome Biol. 2021 Jan 19;22:24. doi: 10.1186/s13059-020-02220-y (PMC7814737; doi:10.1186/s13059-020-02220-y)
Supplement: Supplementary file 3 — Additional file 3. Review history. [file 13059_2020_2220_MOESM3_ESM.docx]

**1^st^ round**

**Reviewer 1**

\# Summary of the paper (motivation, methods, results) and general comments

The authors proposed a deep learning method, M2A, for learning higher order features from DNA methylation data that would be predictive of promoter activity: they focus mostly on predicting H3K27ac and H3K4me3 enrichment. The authors argue and expect that methylation patterns can accurately predict histone modifications (i.e. predict ChIP-seq data), which in turn can be informative in terms of gene expression activity. Using a variety of datasets, the authors show that M2A can accurately predict HM enrichment across tumour types (even being trained on a subset of tumours) and also can be used to predict tumour type-specific promoter usage for genes with multiple promoters. Although the paper is well written, I believe the authors (1) should have used a baseline model to show how much additional predictive power one gains by using a DL approach, and (2) should have focused more on explaining/understanding the model output, in terms of which methylation patterns are predictive of HMs.

\# Major comments

1. Although the authors focus on applying M2A across diverse datasets, they do not adequately explain the M2A model assumptions/choices in the main text. For instance, I believe it should be made clear in the main text that M2A uses a relatively large region around +/- 25 kb window around each TSS to predict HM activity for a narrower window +/-2 kb around TSS. This could be explained in Fig2, however, the Figure legend (for this and other figures) is not that informative. Regarding M2A architecture, the authors claim to model the spatial information of neighbouring CpGs, however, in the methods section it seems they do not directly model individual CpG information and their locations across the genome. On the contrary, they use summary window level statistics (such as mean and variance), implying that spatial information is captured only between sliding windows? Finally, focusing on the summary statistics extracted to be used as input features to M2A, aren't the variance and sum of squared differences features highly correlated? Why would the authors believe that this might contain additional predictive information? And if yes, how would then the authors be able to interpret the output of M2A in terms of predictive features that might have some biological relevance?

2. The authors did not compare M2A to any baseline method. This would allow us to quantify the additional predictive power the deep learning model provides (e.g. by extracting potential spatial correlation of methylation patterns). For instance, could the authors use a simple baseline, e.g. by taking the window averages (the 20 250 bp sliding windows around TSS) as input features, and potentially adding the number of CpGs as additional feature (see PMID: 32433972) and apply a vanilla Random Forest or SVM regression model to predic HM enrichment?

3. We thank the authors for making their code publicly available on github. From the github page I could not find any accompanying code to reproduce the figures/results shown in the manuscript. For reproducibility purposes it would be desirable to provide all details/code the authors used in the paper. Although I understand these details could be added after the manuscript has passed the review process. In addition, I have some questions regarding the large R^2 values (see my next comment) obtained by the authors which seem rather high when compared with existing studies.

4. Could the authors explain, how R^2 is computed and also provide online code for their analysis (for reproducibility purposes). The R^2 values for both predicting HM enrichment and showing association with gene expression levels seem rather high. Reading the papers the authors compared their R^2 values in the Discussion section, it seems they just compute R^2 as the square of Pearson's correlation. These are the Kapourani (2016, PMID: 27587656) and Dong (2012, PMID: 22950368), which mostly show Pearsons's r between predicted and observed expression values. Since the authors did not perform similar pre-processing steps (e.g. keeping the same genes, same normalisation of HMs and window size, etc), I am not sure if the comparison of the R^2 is that meaningful and potentially might be misleading, as I explain below.

- For instance, focusing on the Dong (2012) study: Using M2A to predict H3K27ac activity, the authors obtain indirect prediction of gene expresion around R^2 = 0.62, i.e. correlation r around 0.8. And this is by just correlating the predicted H3K27ac activity (x-axis) with the log2(FPKM) from RNA-seq (y-axis). Looking at the Dong (2012) paper, the authors used a large number of HMs, plus additional features and achieved best performance in terms correlation, around 0.8 (Dong Fig 3 RNA-seq experiments). And to achieve this, they used a random forest classifier (to predict expressed vs non-expressed genes) in combination with the linear regression model to predict actual gene expression levels. And importantly, they showed that H3K27ac could explain a small fraction of expression variability and other HMs were more predictive (Dong Fig 2). So my question then is, how can the authors obtain that high R^2, by using a model that is not trained to predict gene expression but H3K27ac activity? From Table S7, the authors show that R^2 = 0.62 when correlating observed K27ac levels with gene expression. Unless I am missing something, there seems to be a discrepancy here, and these values should not be compared with the existing studies, unless a direct comparison is made with the same pre-processing criteria. The same also holds for the other comparisons made in the Discussion section. My main concern is that the way these results are present implicate that only from DNAm patterns one can predict not only all HM activity, but also the corresponding gene expression levels (and only from one HM), without the need to perform ChIP-seq. Although this would be remarkable, demonstrating the rich information content present in DNAm, I don't think it is backed up without direct comparison with those studies.

5. Although M2A is a method that learns some representation of DNAm patterns to predict HM activity, by reading the paper I felt the focus (and interpretation) was mostly on the predicted HM enrichment and its association with expression, instead of explaining what type of higher level features are extracted from M2A, and which are predictive of promoter activity. I believe trying to interpret some of the higher level features learnt from M2A and their consistency in predicting promoter activity would boost the manuscript.

- For instance in Fig 4, gene GAS2, could the authors explain how and why M2A predicted high activity for ERMS sample and low H3K27ac activity for ARMS, given that promoter region around TSS is unmethylated in both samples? In other words, which parts of the met pattern in Fig 4b, were predictive of high HM activity?

- In Fig 5, the authors could have added the methylation track as well and potentially explain the differences in promoter activity and expression?

- In SFig 6b-c and Table S8, there were many samples that actual vs predicted HM activity was really low, sometimes even around R^2 = 0.07. Here, the authors claim that some of these ChIP-seq experiments were of low quality. And potentially this is the case, because the predicted H3K27ac activity had higher correlation with gene expression levels compared to actual values. However, what if for the specific samples, H3K27ac was not that correlated with gene expression, e.g. other HMs might be more predictive? Could the authors show some patterns of DNAm which generally would result in high HM activity, but this was not the case for some of those 'poor' ChIP-seq samples?

\# Minor comments

1. Lines 80 and 99: The authors mention the lack of interpretability of DNA met patterns and M2A would allow to functionally interpret it. Reading the paper it was not shown how M2A can be used to interpret the DNA methylation patterns, only that it can accurately predict HM enrichment. Maybe these sentences need to be re-written to make this point more clear?

2. Figure captions are not informative enough to extract content from each figure. For instance in Fig 1, there is no label in the colour bar at the top of panels a,b and c. Also are the heatmaps only for H3K27ac activity? The +,- signs at the left of each heatmap should be explained. What do the values in the Venn diagram denote? I know some information could be extracted, but it would help the reader if the figures were more self contained.

3. Line 139: Already other methods, such as BPR (PMID: 27587656) and DeepCpG (PMID: 28395661), extract higher order methylation features, albeit for different applications than predicting promoter activity. Maybe this sentence needs to be re-written to acknowledge existing work.

4. Fig 2: Essentially Fig2b is a zoom version of Fig 2a on right panel. Not sure how much information content this adds.

5. Lines 171-174: Not clear what the authors mean here. Table S3 contains the R^2/RMSE between observed and predicted values, however, not sure what the following statement means: M2A outperformed the observed consistency between training and testing samples?

6. Lines 319-320 regarding Figure 4a: If authors showed PCA (first 2 PCs) instead of t-SNE, wouldn't the samples split by tumour type? Also, wouldn't this finding then imply that the subtype-specific epigenetic regulation is also present in the methylation patterns? For example if the authors just performed a simple PCA of the samples based on mean methylation levels, wouldn't the samples already show a clear separation between ARMS and ERMS?

7. Figure 5: This Figure is poorly explained in the caption. Also there is no PDZRN3-205 isoform show in the figure, but explained in the text. The DZRN3-001 is not mentioned in the text and not explained why it is show in the figure. Also, I am not sure how informative this plot is, since it shows the expression and HM activities across the subtypes (for two samples), but it does not show and explain what differences in methylation patterns drove M2A in predicting the existence of subtype-specific promoter usage. So could the authors also show the methylation levels track as well here?

\# GBIO questions

- Are the methods appropriate to the aims of the study, are they well described, and are necessary controls included?

Mostly yes, although additional explanantion of the model choices and a high level introduction in the main text would make the paper stronger.

- Are the conclusions adequately supported by the data shown?

The statements made in the Discussion section are not supported if comparisons are not made with the same processing steps as stated in my comments above.

- Are sufficient details provided to allow replication and comparison with related analyses that may have been performed?

A high level description of pre-processing steps is provided. However, for reproducibility purposes, it would be desirable if the authors uploaded their analysis scripts on Github as well.

- Is the method likely to be of broad utility? Is any software component easy to install and use? Please indicate briefly the novel features and/or advantages of the method, and/or please reference the relevant publications and which methods, if any, it should be compared with.

The method will likely find adoption in the community, since it claims to acurately predict promoter acticity from DNAm assays. The method requires common software components that are often used in deep learning, and the Github page clearly explains the steps to run M2A.

- Is the paper of broad interest to others in the field, or of outstanding interest to a broad audience of biologists?

Yes.

**Reviewer 2**

Williams et al. have designed and implemented neural networks to predict two histone modifications (H3K4me3 and H3K27ac -- one neural network per histone modification) from CpG methylation in whole bisulfite-converted genomes of pediatric tumors.

For this reviewer, the strongest aspects of this study are the importance of the problem being addressed - essentially, imputing one type of epigenetic mark from another more readily investigated one, the overall clarity of the writing, and at a technical level the authors’ diligence in evaluating their machine learning model across several methyl-seq plus ChIP-seq datasets. There are several areas, some quite fundamental, where substantive improvements need to be made because this reviewer is not yet fully convinced of the utility of the approach.

Major criticisms and suggestions:

1. The authors present a title and an abstract suggesting that they were able to infer promoter activity from CpG methylation, when a more precise and accurate description would be that they were able to infer two histone modifications from CpG methylation. Promoter activity correlates with the two histone modifications they studied but there are other histone modifications influencing promoter activity and all their contributions are probably coupled (Roadmap Epigenomics Consortium et al. 2015). Perhaps as a result, they only find a R-squared of about 0.60 between their predictions and the actual promoter activity (as measured by gene expression). The manuscript could be strengthened by incorporating additional chromatin features – to substantively improve this situation.

2. We already knew that these histone modifications very strongly correlate with methylation (approx. -0.7 for H3K27ac and approx. -0.35 for H3K4me3) (Sharifi-Zarchi et al. 2017). Neural networks are useful when a multitude of features, each individually weakly correlating with the labels, non-linearly contribute together to explain the labels. Here, there is such a strong association between methylation and histone modification that the usefulness of a neural network approach might even be somewhat overstated. If the authors believe otherwise, it would be great to see a side-by-side comparison between a prediction relying on a linear regression and their neural network. In other words, while the authors compared the performance of a traditional artificial neural network model consisting of only two FC layers to that of a deep-learning framework with three CNN layers and two FC layers, they did not do a more basic comparison of their approach with a linear regression relying on machine-learning. It is crucial that this problem with usefulness and significance and redundancy be addressed – see point #1 above – which also requests substantive improvements, perhaps by adding more features to the analysis.

3. Important: the Github repository should include examples of inputs for the model.

4. Why not make the fully-trained model available to researchers on Github? That way, they can use its predictive power without having to train it.

5. The figures about the output of the model are difficult to understand without referring to the legends, and there is also some waste of space with diagrams that are overly generic. These figures need to be made clearer. This point might seem minor – but in fact it is major – as one example – Figure 1a shows visually striking images, but what does (+,+,+) or (-,-,+) mean? Also, what are the subsets in the Venn diagrams in Fig 1 – these are likewise not labeled. Fig. 2B is a generic image of a neural network -- not sure what it brings to the paper. What does the color scale represent in Figure 3 c-g? Why not plot the individual data points? In Fig. 4a, the meaning of the x- and y-axes is not evident from the figure itself, although information is given in the text and legend. Most of the axis scales are indicated in tiny font that cannot be read without zooming in. Every figure should be re-made so that it can be basically understood without having to read the legends.

6. The authors should provide a Docker image with all the required packages for their model so that people interested in testing / using their tools can do it more easily.

Minor comments:

Line 88: the authors should specify here, up-front not only in the following sections, the platforms utilized for DNA methylation profiling (e.g. WGBS, etc.).

Refs:

Roadmap Epigenomics Consortium, Anshul Kundaje, Wouter Meuleman, Jason Ernst, Misha Bilenky, Angela Yen, Alireza Heravi-Moussavi, et al. 2015. “Integrative Analysis of 111 Reference Human Epigenomes.” Nature 518 (7539): 317–30.

Sharifi-Zarchi, Ali, Daniela Gerovska, Kenjiro Adachi, Mehdi Totonchi, Hamid Pezeshk, Ryan J. Taft, Hans R. Schöler, et al. 2017. “DNA Methylation Regulates Discrimination of Enhancers from Promoters through a H3K4me1-H3K4me3 Seesaw Mechanism.” BMC Genomics 18 (1): 964.

**Reviewer 3**

In this manuscript, the authors proposed a deep learning-based method, M2A, for prediction of promoter activity using the DNA methylation data around TSS. The topic is very significant since ChIP-seq data are not always available and an accurate method as such can be used to infer the missing histone modification (HM) data. The inferred HM data could offer potential mechanistic insights as well as be used in downstream analysis tasks such as constructing diagnostic and prognostic models. Although the computational method used is not novel, the application to the DNA methylation data is considered to be original. The evaluation of the method is also thorough and convincing, and supports their major conclusions. Following are my comments/suggestions the authors may consider in improving their manuscript.

1. The method belongs to the topic of gene activity prediction based on DNA sequence, structure and other upstream events. A brief review of the relevant literature will put their approach into the right context.

2. Study the impact of model parameters on the performance. It seems the model parameters were all preset. It will be informative how the key parameters influence the model performance. For example, the DNA length used is an important parameter. The optimal length itself is very interesting.

3. Some naïve method such as fitting a spline model based on the average methylation around TSS can be compared to have a clearer view of the actual improvement.

4. M2A is based solely on the methylation data. Can the DNA sequence data be leveraged to increase the prediction accuracy?

5. Two HM markers were investigated in this study. How is the performance for other markers? .

6. The evaluation plan can be better illustrated by some flow chart.

**Author response**

The authors proposed a deep learning method, M2A, for learning higher order features from DNA methylation data that would be predictive of promoter activity: they focus mostly on predicting H3K27ac and H3K4me3 enrichment. The authors argue and expect that methylation patterns can accurately predict histone modifications (i.e. predict ChIP-seq data), which in turn can be informative in terms of gene expression activity. Using a variety of datasets, the authors show that M2A can accurately predict HM enrichment across tumour types (even being trained on a subset of tumours) and also can be used to predict tumour type-specific promoter usage for genes with multiple promoters. Although the paper is well written, I believe the authors (1) should have used a baseline model to show how much additional predictive power one gains by using a DL approach, and (2) should have focused more on explaining/understanding the model output, in terms of which methylation patterns are predictive of HMs.

**Response:** We thank the reviewer for appreciating the potential impact of the proposed method. We have followed his/her suggestions to include additional baseline models and improved the presentation on the understanding of the model outputs in the revised manuscript. We believe we have fully addressed the reviewer’s concerns.

**Major comment**

1.1) Although the authors focus on applying M2A across diverse datasets, they do not adequately explain the M2A model assumptions/choices in the main text. For instance, I believe it should be made clear in the main text that M2A uses a relatively large region around +/- 25 kb window around each TSS to predict HM activity for a narrower window +/-2 kb around TSS. This could be explained in Fig2, however, the Figure legend (for this and other figures) is not that informative.

**Response:** We apologize that we did not adequately explain the key assumptions for M2A. We followed the suggestion and explain the model specification in the revised manuscript. Moreover, we revised the figure legends throughout the manuscript.

In the revision:

“The central hypothesis of M2A is that the complex relation between DNAm signatures and promoter activities (measured as H3K4me3 and H3K27ac enrichment in the TSS ± 1 kb region) can be captured by incorporating both summary statistics extracted from window-based CpG methylation levels and high-order spatial information from these windows in the promoter and flanking regions (up to 25 kb from the TSS).”

1.2) Regarding M2A architecture, the authors claim to model the spatial information of neighbouring CpGs, however, in the methods section it seems they do not directly model individual CpG information and their locations across the genome. On the contrary, they use summary window level statistics (such as mean and variance), implying that spatial information is captured only between sliding windows?

**Response:** We agree with the reviewer’s interpretation and have clarified that the spatial information was captured between sliding windows (instead of individual CpGs) in the revised manuscript.

In the revision:

“The central hypothesis of M2A is that the complex relation between DNAm signatures and promoter activities (measured as H3K4me3 and H3K27ac enrichment in the TSS ± 1 kb region) can be captured by incorporating both summary statistics extracted from window-based CpG methylation levels and high-order spatial information from these windows in the promoter and flanking regions (up to 25 kb from the TSS).”

1.3) Finally, focusing on the summary statistics extracted to be used as input features to M2A, aren't the variance and sum of squared differences features highly correlated? Why would the authors believe that this might contain additional predictive information? And if yes, how would then the authors be able to interpret the output of M2A in terms of predictive features that might have some biological relevance?

**Response:** We thank the reviewer for pointing out the close relation between these two raw input features. By incorporating both features, we inexplicitly encode the number of CpGs captured in each window. Moreover, we consider the number of CpGs is a (relatively) static feature shared among different samples for the same promoter. In the over-parameterized deep-learning models, including such static features raises the concern of model overfitting by potentially memorizing the genomic position/identity of the promoter. We tested the model performance after removal of the sum of squared differences (SSD) feature. Our extensive evaluation suggested that the new model with the remaining three raw input features achieved comparable performance with the initial model (Additional file 2: Table S2). Consequently, we have updated the feature design to exclude the SSD features and rerun the entire analyses. The new model was presented in the revision manuscript and in the Github repository and the Docker image.

In the revision:

“The DNA methylation features, including the windowed M-value mean, variance, and the fraction of the SSD of M-values (FSSD), were calculated and represented by the feature vectors $\boldsymbol{Mave}_{\boldsymbol{i}}$, $\boldsymbol{Mvar}_{i}$, and $\boldsymbol{Mfssd}_{i}$.”

2)	The authors did not compare M2A to any baseline method. This would allow us to quantify the additional predictive power the deep learning model provides (e.g. by extracting potential spatial correlation of methylation patterns). For instance, could the authors use a simple baseline, e.g. by taking the window averages (the 20 250 bp sliding windows around TSS) as input features, and potentially adding the number of CpGs as additional feature (see PMID: 32433972) and apply a vanilla Random Forest or SVM regression model to predict HM enrichment?

**Response:** We followed the reviewer’s suggestion and included two additional baseline models (together with the ANN model with 2 FC layer presented in the initial submission): a vanilla Random Forest model (implemented by scikit-learn in python) and a multivariate adaptive regression splines model (MARS model, implemented by the R “earth” package) using the identical input features (3 features, 2 resolutions and 20 windows). The result was presented in Additional file 2: Table S2. In summary, the deep-learning model implemented in M2A outperformed all three baseline models for both H3K4me3 and H3K27ac prediction in the NBL validation dataset with 10 samples.

Moreover, although we decided to exclude the number of CpG information in our M2A design, we nevertheless tested additional Random Forest and MARS models for H3K27ac by including the number of CpG per window as additional features. The addition of the number of CpG feature indeed decreased the performance in the validation set for both random forest and MARS models, which is consistent with our concern that the static feature may lead into overfitting (See Appendix Table 1 and Appendix Table 2).

In the revision:

“Using the validation set, we compared the performance of the M2A framework of three CNN layers and two FC layers (Figure 2) with three frequently used statistical and machine learning approaches (baseline models), namely multivariate adaptive regression splines (MARS), random forest, and artificial neural network (ANN) consisting of only two FC layers. In every instance, the M2A framework outperformed baseline models (Additional file 2: Table S2). From a qualitative perspective, M2A correctly revealed the bimodal distribution of the promoter activities for both H3K4me3 and H3K27ac in all samples, and from a quantitative perspective, the inferred genome-wide promoter activity landscape was highly accurate for individual samples for both H3K4me3 (*R*^2^ = 0.933 ± 0.019; RMSE = 0.621 ± 0.072) (Figures 3a and 3d) and H3K27ac (*R*^2^ = 0.799 ± 0.053; RMSE = 0.644 ± 0.074) (Figures 3b, and 3c). Moreover, the addition of CNN layers was merited, as there was a decrease in the prediction error (measured as 1 − *R*^2^) from the next highest performer by 17.8% (*P* = 0.0020, Wilcoxon signed-rank test) and 12.4% (*P* = 0.0020, Wilcoxon signed-rank test) for the model topologies for H3K4me3 and H3K27ac, respectively (Additional file 2: Table S2).”

3)	We thank the authors for making their code publicly available on github. From the github page I could not find any accompanying code to reproduce the figures/results shown in the manuscript. For reproducibility purposes it would be desirable to provide all details/code the authors used in the paper. Although I understand these details could be added after the manuscript has passed the review process. In addition, I have some questions regarding the large R^2 values (see my next comment) obtained by the authors which seem rather high when compared with existing studies.

**Response:** We thank the review for his/her suggestion. We have included the code used to generate all major figures to the github page.

4)	Could the authors explain, how R^2 is computed and also provide online code for their analysis (for reproducibility purposes). The R^2 values for both predicting HM enrichment and showing association with gene expression levels seem rather high. Reading the papers the authors compared their R^2 values in the Discussion section, it seems they just compute R^2 as the square of Pearson's correlation. These are the Kapourani (2016, PMID: 27587656) and Dong (2012, PMID: 22950368), which mostly show Pearsons's r between predicted and observed expression values. Since the authors did not perform similar pre-processing steps (e.g. keeping the same genes, same normalisation of HMs and window size, etc), I am not sure if the comparison of the R^2 is that meaningful and potentially might be misleading, as I explain below.

For instance, focusing on the Dong (2012) study: Using M2A to predict H3K27ac activity, the authors obtain indirect prediction of gene expresion around R^2 = 0.62, i.e. correlation r around 0.8. And this is by just correlating the predicted H3K27ac activity (x-axis) with the log2(FPKM) from RNA-seq (y-axis). Looking at the Dong (2012) paper, the authors used a large number of HMs, plus additional features and achieved best performance in terms correlation, around 0.8 (Dong Fig 3 RNA-seq experiments). And to achieve this, they used a random forest classifier (to predict expressed vs non-expressed genes) in combination with the linear regression model to predict actual gene expression levels. And importantly, they showed that H3K27ac could explain a small fraction of expression variability and other HMs were more predictive (Dong Fig 2). So my question then is, how can the authors obtain that high R^2, by using a model that is not trained to predict gene expression but H3K27ac activity? From Table S7, the authors show that R^2 = 0.62 when correlating observed K27ac levels with gene expression. Unless I am missing something, there seems to be a discrepancy here, and these values should not be compared with the existing studies, unless a direct comparison is made with the same pre-processing criteria. The same also holds for the other comparisons made in the Discussion section. My main concern is that the way these results are present implicate that only from DNAm patterns one can predict not only all HM activity, but also the corresponding gene expression levels (and only from one HM), without the need to perform ChIP-seq. Although this would be remarkable, demonstrating the rich information content present in DNAm, I don't think it is backed up without direct comparison with those studies.

**Response:** R^2^ is calculated as the square of Pearson correlation coefficient.

To evaluate whether difference in the data preprocessing and gene/promoter filtering may affect the calculate the R2 performance, we followed the filtering steps described in Dong et al. (2012) and updated relevant RNA-seq expression based analyses in the test datasets of RMS, ENCODE and BLUEPRINT-AML. For ENCODE samples, the FPKM values were downloaded from ENCODE data portal <https://www.encodeproject.org/> . M2A achieved a comparable performance with the new preprocessing (average R^2^ = 0.668, Supplementary Table S7), which further supported the predictive value of M2A model in gene expression. Since M2A takes only DNAm features, we don’t think other preprocessing steps for HM-based features affect our analysis.

We caution that Dong (2012) evaluated the feature importance in a multivariate prediction model, which included highly correlated histone marks (such as H3K9ac and H3K27ac). Karlić et al. (2010) showed that H3K27ac level generated the best single-mark linear regression model (Pearson’s r=0.72) in predicting expression among 41 chromatin features. Moreover, the full model with all 41 marks only achieved a slightly better performance (r=0.77), which suggested a high degree of redundancy in these chromatin features. Consequently, Karlić et al. concluded that “the levels of a single modification(H3K27ac) can be used to faithfully model gene expression”. Combining both our results and the result from Karlić et al., we believe that by accurately predicting H3K27ac levels from DNAm patterns, we are able to model gene expression levels as well.

Reference:

Karlić R, Chung H-R, Lasserre J, Vlahovicek K, Vingron M: Histone modification levels are predictive for gene expression. Proc Natl Acad Sci USA. 2010, 107: 2926-2931. 10.1073/pnas.0909344107.

In the revision:

“The correlation between gene expression and inferred promoter activities (mean *R*^2^ = 0.668 for ENCODE data, 0.722 for K562, 0.705 for GM12878, and 0.536 for H1-ESC) surpassed that with the state-of-art BPR model [42], which was developed for predicting gene expression levels from DNAm patterns (the best reported *R*^2^ values were 0.49 for K562, 0.37 for GM12878, and 0.25 for H1-ESC). Strikingly, the (indirect) predictive accuracy of M2A for gene expression across nine ENCODE cell lines (average *R*^2^ = 0.668) was comparable to the predictive accuracy of a model built on 11 HMs, one histone variant, and DNase I hypersensitivity [11]. Similarly, the accuracy of binary prediction of expressed genes (average AUC = 0.941 and 0.931 for the ENCODE and AML datasets, respectively) surpassed that of DeepChrome (average AUC = 0.80), a state-of-art deep-learning algorithm trained to predict expressed genes by using five core HMs (H3K4me3, H3K4me1, H3K36me3, H3K9me3, and H3K27me3) [12].”

5.1)	Although M2A is a method that learns some representation of DNAm patterns to predict HM activity, by reading the paper I felt the focus (and interpretation) was mostly on the predicted HM enrichment and its association with expression, instead of explaining what type of higher level features are extracted from M2A, and which are predictive of promoter activity. I believe trying to interpret some of the higher level features learnt from M2A and their consistency in predicting promoter activity would boost the manuscript.

For instance in Fig 4, gene GAS2, could the authors explain how and why M2A predicted high activity for ERMS sample and low H3K27ac activity for ARMS, given that promoter region around TSS is unmethylated in both samples? In other words, which parts of the met pattern in Fig 4b, were predictive of high HM activity?

**Response:** We agree with Reviewer 1 that an in-depth analysis of feature performance would be beneficial to the manuscript. Interpretation of high-level features extracted from CNN remains as an active research topic and is generally considered as an unsolved problem (Barredo et al., 2020). Nevertheless, we included two additional analyses to understand why M2A achieves high consistency in predicting promoter activates. First, we analyzed the distribution of raw input features. We start by classified promoters in individual samples three categories: active (high H3K27ac), poised (high H3K4me3 and low H3K27ac) and inactive (low H3K4me3 and low H3K27ac). Then we compared the average (and 95% confidence interval) curve along the sliding windows for individual raw input features. The analysis revealed that the overall shape for individual features is more discriminative than average DNAm values in the promoter regions in distinguishing the three categories, which provide the feasibility that CNNs can extract generalized high-order features to predict the response variable. For example, the average M-val feature in the 2.5 kb window demonstrated that while both active and poised promoters have hypomethylation near TSS, poised promoters tend to have large partially methylated pattern further away from the TSS, which may lead to increased H3K27me3 levels in the entire regions.

In the second analysis, we compared the correlation between individual features and the response variable (H3K27ac) for both the raw input features and the CNN extracted features. The analysis clearly indicated that the CNN extracted high-order feature is highly correlated with the response variable compared to the raw input features, which partly explain the performance gain for the deep-learning based M2A model compared to the baseline models.

For example, although the average methylation level surrounding TSS of the *GAS2* promoter is comparable between ARMS and ERMS samples, when we expand the region further to both 5’ and 3’ ends, we observe that ERMS samples showed hypermethylation in the gene body (signature for actively transcribed gene body) while ARMS samples showed reduced methylation levels, which is consistent with the partially methylated domain, a typically repressive domain.

Reference:

Barredo Arrieta A, Díaz-Rodríguez N, Del Ser J, Bennetot A, Tabik S, Barbado A, Garcia S, Gil-Lopez S, Molina D, Benjamins R, et al: Explainable Artificial Intelligence (XAI): Concepts, taxonomies, opportunities and challenges toward responsible AI. Information Fusion. 2020, 58:82-115.

In the revision:

“We started with an analysis of the information content in DNAm patterns by examining the input feature distribution in different windows, among active (high H3K27ac), poised (high H3K4me3 and low H3K27ac), and inactive promoters (low H3K4me3 and low H3K27ac) in the six NBL O-PDX training samples. These features show distinct patterns among the three promoter categories (Additional File 1: Figure S2), indicating the feasibility of modeling promoter activities from DNAm patterns. Although the interpretability of CNN extracted features remains an active field of research in deep-learning [54] we examined the efficacy of CNN extracted features in modeling the promoter activities. We first compared the square of Pearson’s correlation (R^2^) between each feature (both raw input and CNN extracted features) and the response variable (H3K27ac) in the training set, The analysis revealed that CNN-extracted features have significantly higher R^2^ with the response (250 bp: *P* = 1.5 × 10^−11^*,* 2500 bp *P* = 3.9 × 10^−5^, Wilcoxon signed-rank test)(Additional File 1: Figure S3)We further evaluated the best features for both raw input and CNN-extracted features in the validation samples and again the CNN-extracted features significantly outperformed the raw input features (250 bp: *P* = 1.1 × 10^−5^*,* 2500 bp *P* = 1.1 × 10^−5^, Wilcoxon signed-rank test)(Additional File 1: Figure S3).”

5.2)	In Fig 5, the authors could have added the methylation track as well and potentially explain the differences in promoter activity and expression?

**Response:** We thank the reviewer for the suggestion and included the DNAm track in Fig. 5.

5.3)	In SFig 6b-c and Table S8, there were many samples that actual vs predicted HM activity was really low, sometimes even around R^2 = 0.07. Here, the authors claim that some of these ChIP-seq experiments were of low quality. And potentially this is the case, because the predicted H3K27ac activity had higher correlation with gene expression levels compared to actual values. However, what if for the specific samples, H3K27ac was not that correlated with gene expression, e.g. other HMs might be more predictive? Could the authors show some patterns of DNAm which generally would result in high HM activity, but this was not the case for some of those 'poor' ChIP-seq samples?

**Response:** We agree with the reviewer that we could not rule out the possibility that in a set of samples, H3K27ac is not strongly correlated with gene expression for a specific biological mechanism. Therefore, we have discussed this possibility in the revised manuscript. Additionally, we analyzed ENCODE data with replicate ChIP-seq experiments and demonstrated that the replicates with better consistency between actual H3K27ac level and predicted H3K27ac level also have higher consistency between actual H327ac level and actual expression level, which supported library quality as a potential interpretation. Moreover, we showed an example of *PSMA7*, which has promoter hypometylation, strong expression in H1-ESC. However, one ChIP-seq experiment showed strong H3K27ac enrichment but the other replicate showed almost no H3K27ac signal (Additional file1: Figure S8d).

In the revision:

“Furthermore, joint analyses with RNA-seq data from the matching samples indicated that 1) in contrast to the predictive accuracy of H3K27ac levels, the “poor performers” achieved comparable consistency between the M2A-inferred promoter activity and gene expression; 2) the “poor performers” showed significantly lower correlation between the measured promoter H3K27ac level and gene expression; 3) the correlation between the measured promoter H3K27ac level and gene expression was highly predictive of the accuracy of the H3K27ac prediction (Additional file 1: Figure S10),and 4) in ENCODE samples with replicates, the replicate with better consistency between H3K27ac and gene expression also showed significantly higher correlation between the actual and measured H3K27ac level (*P =* 0.0039, Wilcoxon signed rank test, Additional file 2: Table S15 an example of H3K27ac signal discrepancy between H1-ESC replicates shown in Additional file1: Figure S8d). Although we cannot unequivocally rule out the possibility that these “poor performers” share a distinct biological mechanism where promoter H3K27ac level is no longer a stronger predictor for gene activities, these results suggested that the “poor performers” could reflect the quality of the ChIP-seq results included in the test data.”

**Minor comment**

1) Lines 80 and 99: The authors mention the lack of interpretability of DNA met patterns and M2A would allow to functionally interpret it. Reading the paper it was not shown how M2A can be used to interpret the DNA methylation patterns, only that it can accurately predict HM enrichment. Maybe these sentences need to be re-written to make this point more clear?

**Response:** We apologize for the confusion. M2A is designed to use DNAm pattern (a reliable epigenome platform for clinical data) to infer levels of two major histone modifications in the promoter regions, with better biological interpretability (transcriptional active promoters carry strong H3K27ac/H3K4me3). We have clarified the design in the revision.

2) Figure captions are not informative enough to extract content from each figure. For instance in Fig 1, there is no label in the colour bar at the top of panels a,b and c. Also are the heatmaps only for H3K27ac activity? The +,- signs at the left of each heatmap should be explained. What do the values in the Venn diagram denote? I know some information could be extracted, but it would help the reader if the figures were more self-contained.

**Response:** We thank the reviewer for the critique. We have updated Figure legends throughout the manuscript to provide additional information.

3) Line 139: Already other methods, such as BPR (PMID: 27587656) and DeepCpG (PMID: 28395661), extract higher order methylation features, albeit for different applications than predicting promoter activity. Maybe this sentence needs to be re-written to acknowledge existing work.

**Response:** We appreciate the suggestion and have acknowledged the existing literatures in the revision.

In the revision.

“Several studies [10-12] have demonstrated that HMs and other epigenetic features can be used to predict gene expression. Using a linear regression model, Karlić et al. show that approximately 50%–60% of the variation in gene expression can be accounted for, and that ~50% of the variation in gene expression can be modeled by promoter H3K27ac enrichment alone [10]. Subsequent work by Dong et al. further explained 69% of gene expression variance using a hybrid random forest/linear regression model with features derived from 11 HMs, one histone variant, and DNase I hypersensitivity [11]. More recently, Singh et al. used deep-learning models on five HMs to predict gene expression status (high/low) and achieved an average AUC of 0.80 [12].”

“However, the highly complex and non-linear relations between DNAm patterns and HMs severely hamper the interpretability of the biological impact of differential DNAm patterns. Previous studies have shown the usefulness of extracting higher-order methylation features [42], for predicting gene expression. Moreover, recent studies applied deep-learning approaches to infer DNAm states from their local sequence composition and adjacent DNAm states [53]. We hypothesize that these high-level DNAm features (that capture the spatial information from DNAm patterns in the promoter and regions in its vicinity) could also provide an opportunity to infer promoter activities such as H3K27ac and H3K4me3 enrichment accurately.”

4) Fig 2: Essentially Fig2b is a zoom version of Fig 2a on right panel. Not sure how much information content this adds.

**Response:** We removed the redundant panel.

5) Lines 171-174: Not clear what the authors mean here. Table S3 contains the R^2/RMSE between observed and predicted values, however, not sure what the following statement means: M2A outperformed the observed consistency between training and testing samples?

**Response:** We apologize for the confusion. Due to the sparsity of ChIP-seq experiments in primary tumors, surrogate model is a common practice for epigenetic analysis in biological/biomedical research, which uses a few epigenomes profiled in model systems (cell lines, PDX, organoids, etc) from the same disease to represent the patient tumor epigenome. In this analysis, we are comparing the M2A’s prediction accuracy in 10 NBL samples in the validation set to the consistency between the 10 samples in validation set and 6 NBL O-PDX models in the training set. Our result demonstrated that even though both training and test samples are NBL models, M2A prediction significantly outperform the surrogate model. We have included the consistency between the training and test NBL samples to Supplementary Table 3).

In the revision:

“a common practice in deciphering major HM deregulations in various cancers is to extrapolate the epigenetic profiles from related cancer models (surrogate models).”

“Our analysis of *MYCN*-amplified NBL cell line and O-PDX models has revealed substantial variations in their promoter activities, which is a potential caveat to the practice of surrogate model (representing primary tumor epigenomes by a few profiled models). Conversely, M2A produced highly accurate promoter activity landscapes, significantly outperforming the observed consistency between training and testing samples for both H3K4me3 (*R*^2^ = 0.891 ± 0.023, *P* = 2.3 × 10^−5^, Wilcoxon rank-sum test) and H3K27ac (*R*^2^ = 0.720 ± 0.045, *P* = 9.5 × 10^−5^, Wilcoxon rank-sum test; Additional file 2: Table S3).”

6) Lines 319-320 regarding Figure 4a: If authors showed PCA (first 2 PCs) instead of t-SNE, wouldn't the samples split by tumour type? Also, wouldn't this finding then imply that the subtype-specific epigenetic regulation is also present in the methylation patterns? For example if the authors just performed a simple PCA of the samples based on mean methylation levels, wouldn't the samples already show a clear separation between ARMS and ERMS?

**Response:** We agree with the reviewer that the subtype-specific epigenome regulation can be measured in both histone modifications and DNAm patterns. PCA analyses using DNAm, actual H3K27ac levels and predicted H3K27ac levels confirmed the hypothesis (Appendix Figure 1). The message we intended to convey here is that the subtype-specific epigenome signature in RMS tumors is faithfully captured in M2A prediction using a model trained on NBL (a different disease), which demonstrates the generalizability of the CNN-extracted high-order DNAm features. We have clarified this point in the revised manuscript.

**Appendix Fig 1** Epigenetic heterogeneity captured by promoter activities (actual and M2A-inferred H3K27ac enrichment) and DNA methylation patterns

In the revision:

“A t-SNE embedding using M2A-inferred promoter activity landscapes (from the NBL-trained model) recapitulated the clear separation of ARMS and ERMS tumors (Figure 4a) in the DNAm profiles (data not shown), which further demonstrates the generalizability of the CNN-extracted high-order DNAm features.”

7) Figure 5: This Figure is poorly explained in the caption. Also, there is no PDZRN3-205 isoform show in the figure, but explained in the text. The DZRN3-001 is not mentioned in the text and not explained why it is show in the figure. Also, I am not sure how informative this plot is, since it shows the expression and HM activities across the subtypes (for two samples), but it does not show and explain what differences in methylation patterns drove M2A in predicting the existence of subtype-specific promoter usage. So could the authors also show the methylation levels track as well here?

**Response:** We followed the suggestion and included the DNAm tracks and improved the figure legend.

**Reviewer 2.**

Williams et al. have designed and implemented neural networks to predict two histone modifications (H3K4me3 and H3K27ac -- one neural network per histone modification) from CpG methylation in whole bisulfite-converted genomes of pediatric tumors.

For this reviewer, the strongest aspects of this study are the importance of the problem being addressed - essentially, imputing one type of epigenetic mark from another more readily investigated one, the overall clarity of the writing, and at a technical level the authors’ diligence in evaluating their machine learning model across several methyl-seq plus ChIP-seq datasets. There are several areas, some quite fundamental, where substantive improvements need to be made because this reviewer is not yet fully convinced of the utility of the approach.

**Response:** We thank the reviewer for appreciating the importance of the proposed method. We have followed his/her suggestions to improve the model and the presentation in the revised manuscript.

**Major comment 1)**

1) The authors present a title and an abstract suggesting that they were able to infer promoter activity from CpG methylation, when a more precise and accurate description would be that they were able to infer two histone modifications from CpG methylation. Promoter activity correlates with the two histone modifications they studied but there are other histone modifications influencing promoter activity and all their contributions are probably coupled (Roadmap Epigenomics Consortium et al. 2015). Perhaps as a result, they only find a R-squared of about 0.60 between their predictions and the actual promoter activity (as measured by gene expression). The manuscript could be strengthened by incorporating additional chromatin features – to substantively improve this situation.

**Response:** We agree with the reviewer that other features, especially feature modeling the enhancer activities and promoter-enhancer interactions will be needed to substantially improve the prediction for gene expression, which are one of our further research topics. However, instead of understanding the relationship between various chromatin features and the transcriptional activity, the primary aim in M2A design is to infer levels of two major histone modifications for promoter activities (H3K4me3 and H3K27ac) from DNAm profiles, the most reliable epigenome platform for clinical samples. Therefore, we purposely designed a low feature profile (all features derived from DNAm profile), which renders the model good portability and generalizability (for example, it can infer H3K4me3/H3K27ac levels in FFPE archived samples). Unfortunately, addition of extra chromatin features will remove M2A’s functionality (if additional chromatin features are available, it is likely RNA-seq, H3K4me3 and H3K27ac can be experimentally profiled).

Moreover, as we discussed in response to Review 1’s Major comment 4, we demonstrated that although M2A is not directly trained on gene expression level, it nevertheless faithfully models the gene expression level and its accuracy is comparable to the performance of top models using a large number of HMs, plus additional features (PMID: 20133639, 22950368).

**Major comment 2)**

2) We already knew that these histone modifications very strongly correlate with methylation (approx. -0.7 for H3K27ac and approx. -0.35 for H3K4me3) (Sharifi-Zarchi et al. 2017). Neural networks are useful when a multitude of features, each individually weakly correlating with the labels, non-linearly contribute together to explain the labels. Here, there is such a strong association between methylation and histone modification that the usefulness of a neural network approach might even be somewhat overstated. If the authors believe otherwise, it would be great to see a side-by-side comparison between a prediction relying on a linear regression and their neural network. In other words, while the authors compared the performance of a traditional artificial neural network model consisting of only two FC layers to that of a deep-learning framework with three CNN layers and two FC layers, they did not do a more basic comparison of their approach with a linear regression relying on machine-learning. It is crucial that this problem with usefulness and significance and redundancy be addressed – see point #1 above – which also requests substantive improvements, perhaps by adding more features to the analysis.

**Response:** We agree with the comments that comparison with additional baseline models is beneficial to demonstrate the usefulness of the deep-learning approach employed in M2A. We tested two additional baseline models: a vanilla Random Forest model (implemented by sckit-learn in python) and a multivariate adaptive regression splines model (MARS model, implemented by the R “earth” package) using the identical input features. Linear regression can be considered as a special form of the MARS model. The results were presented in Supplementary Table X. In summary, the deep-learning model implemented in M2A outperformed all three baseline models for both H3K4me3 and H3K27ac prediction in the validation NBL dataset with 10 samples.

In the revision:

“Using the validation set, we compared the performance of the M2A framework of three CNN layers and two FC layers (Figure 2) with three frequently used statistical and machine learning approaches (baseline models), namely multivariate adaptive regression splines (MARS), random forest, and artificial neural network (ANN) consisting of only two FC layers. In every instance, the M2A framework outperformed baseline models (Additional file 2: Table S2). From a qualitative perspective, M2A correctly revealed the bimodal distribution of the promoter activities for both H3K4me3 and H3K27ac in all samples, and from a quantitative perspective, the inferred genome-wide promoter activity landscape was highly accurate for individual samples for both H3K4me3 (*R*^2^ = 0.933 ± 0.019; RMSE = 0.621 ± 0.072) (Figures 3a and 3d) and H3K27ac (*R*^2^ = 0.799 ± 0.053; RMSE = 0.644 ± 0.074) (Figures 3b, and 3c). Moreover, the addition of CNN layers was merited, as there was a decrease in the prediction error (measured as 1 − *R*^2^) from the next highest performer by 17.8% (*P* = 0.0020, Wilcoxon signed-rank test) and 12.4% (*P* = 0.0020, Wilcoxon signed-rank test) for the model topologies for H3K4me3 and H3K27ac, respectively (Additional file 2: Table S2).”

3) Important: the Github repository should include examples of inputs for the model.

**Response:** We have updated the Github page to include an example of model inputs.

**Major comment 4)**

4) Why not make the fully-trained model available to researchers on Github? That way, they can use its predictive power without having to train it.

**Response:** We have included the fully-trained model for both H3K4me3 and H3K27ac enrichment prediction on Github at (https://github.com/chenlab-sj/M2A/tree/master/6_InputFiles). We have highlighted the model in the ReadMe documentation on the M2A Github repository.

5) The figures about the output of the model are difficult to understand without referring to the legends, and there is also some waste of space with diagrams that are overly generic. These figures need to be made clearer. This point might seem minor – but in fact it is major – as one example – Figure 1a shows visually striking images, but what does (+,+,+) or (-,-,+) mean? Also, what are the subsets in the Venn diagrams in Fig 1 – these are likewise not labeled. Fig. 2B is a generic image of a neural network -- not sure what it brings to the paper. What does the color scale represent in Figure 3 c-g? Why not plot the individual data points? In Fig. 4a, the meaning of the x- and y-axes is not evident from the figure itself, although information is given in the text and legend. Most of the axis scales are indicated in tiny font that cannot be read without zooming in. Every figure should be re-made so that it can be basically understood without having to read the legends.

**Response:** We thank the reviewer for the critic. We have updated Figure legends throughout the manuscript to provide additional information.

In the initial submission:

Figure 1a: The original legend (+,+,+) represents the promoter was active in all 3 models and (-,-,+) represents the promoter is active in the third model only; this has been updated to prevent confusion.

Figure 2b: we have removed the redundant panel.

Figure 3c-g: the color scale in the initial submission represented the log-scaled 2D density. We have updated the figure to plot individual promoters.

**Major comment 6)**

6) The authors should provide a Docker image with all the required packages for their model so that people interested in testing / using their tools can do it more easily.

**Response:** We followed the reviewer’s suggestion and provided a Docker image with all packages pre-loaded (available at: <https://github.com/chenlab-sj/M2A>). To further facilitate potential users, we have implemented a cloud version of M2A, which is available on the St. Jude Cloud (<https://platform.stjude.cloud/workflows/methylation-to-activity>).

**Minor comment**

1) Line 88: the authors should specify here, up-front not only in the following sections, the platforms utilized for DNA methylation profiling (e.g. WGBS, etc.).

**Response:** We agree with reviewer and clarify the platforms used in the study (WGBS and RRBS).

In the revision:

“Using a cohort of six pediatric neuroblastoma (NBL) orthoptic patient-derived xenograft (O-PDX) samples profiled in the Pediatric Cancer Genome Project (PCGP) [43], we trained the model using whole-genome bisulfite sequencing (WGBS) data to predict the enrichment of H3K4me3 and H3K27ac (two HMs critical for promoter [10]) for genome-wide annotated promoters. We validated the predictive accuracy of the model in the remaining NBL samples (N = 10, WGBS) from the same cohort. We further confirmed its accuracy and generalizability in diverse tumor types from four publicly available datasets representing real-world applications, including (1) pediatric rhabdomyosarcoma (RMS) O-PDX tumors profiled in the Pediatric Cancer Genome Project (N = 16, WGBS) [44]; (2) a set of commonly used cell lines profiled in ENCODE (N = 9, WGBS) [45]; (3) primary acute myeloid leukemias (AMLs) profiled by the BLUEPRINT consortium (N = 19, WGBS) [46]; and (4) a large primary Ewing sarcoma (EWS) cohort using reduced representation bisulfite sequencing (RRBS) (N = 140) [22].”

**Reviewer 3.**

In this manuscript, the authors proposed a deep learning-based method, M2A, for prediction of promoter activity using the DNA methylation data around TSS. The topic is very significant since ChIP-seq data are not always available and an accurate method as such can be used to infer the missing histone modification (HM) data. The inferred HM data could offer potential mechanistic insights as well as be used in downstream analysis tasks such as constructing diagnostic and prognostic models. Although the computational method used is not novel, the application to the DNA methylation data is considered to be original. The evaluation of the method is also thorough and convincing, and supports their major conclusions. Following are my comments/suggestions the authors may consider in improving their manuscript.

**Response:** We thank the reviewer for his/her positive comments.

**Major comments**

1) The method belongs to the topic of gene activity prediction based on DNA sequence, structure and other upstream events. A brief review of the relevant literature will put their approach into the right context.

**Response:** We followed the suggestion and added the brief review in introduction.

In the revision:

“Several studies [10-12] have demonstrated that HMs and other epigenetic features can be used to predict gene expression. Using a linear regression model, Karlić et al. show that approximately 50%–60% of the variation in gene expression can be accounted for, and that ~50% of the variation in gene expression can be modeled by promoter H3K27ac enrichment alone [10]. Subsequent work by Dong et al. further explained 69% of gene expression variance using a hybrid random forest/linear regression model with features derived from 11 HMs, one histone variant, and DNase I hypersensitivity [11]. More recently, Singh et al. used deep-learning models on five HMs to predict gene expression status (high/low) and achieved an average AUC of 0.80 [12].”

2) Study the impact of model parameters on the performance. It seems the model parameters were all preset. It will be informative how the key parameters influence the model performance. For example, the DNA length used is an important parameter. The optimal length itself is very interesting.

**Response:** We appreciate the comments on the parameter selection in the deep learning model. Several parameters (kernel size and batch size) were tuned using the NBL samples. Other CNN parameters are set as default in Keras (version:v2.2.4), Following the suggestion of Reviewer 1, we have updated our feature design and re-tuned the parameters (Additional file 2: Table S18).

For DNA length selection, we tested three separate configurations; 1) 100bp+1000bp 2) 250bp+2500bp and 3) 500bp +5000bp. The smaller window size resulted in a substantial fraction of windows lacking sufficient number of CpGs to calculate the features. Using 100bp window, more than 50% of raw input features were missing (Additional file 1: Figure S13) and therefore we did not further evaluate this model. The 250bp+2500bp model outperformed the 500bp+5000bp model (Additional file 2: Table S18).

In the revision:

“**M2A parameter tuning**

Parameters such as the window size, batch size, and kernel size were optimized using the validation NBL data set (n=10). Each parameter configuration was tested holding all other parameters constant, and models with a numerical performance advantage were chosen. For batch size, three configurations were tested (64, 128, 256). Four kernel size configurations ([1,2], [1,3], [1,4], [1,5]) were tested. and window configurations ([100bp, 1000bp], [250bp, 2500bp], [500bp, 5000bp]) were considered. Due to >50% uninformative features in the 100bp window resolution, only the (250bp, 2500bp) model and (500bp, 5000bp) model performances were compared”

3) Some naïve method such as fitting a spline model based on the average methylation around TSS can be compared to have a clearer view of the actual improvement.

**Response:** We followed the suggestion, which is shared among all three reviewers, and tested two additional baseline models: a vanilla Random Forest model (implemented by sckit-learn in python) and a multivariate adaptive regression splines model (MARS model, implemented by the R “earth” package) using the identical input features. The results were presented in Supplementary Table X. In summary, the deep-learning model implemented in M2A outperformed all three baseline models for both H3K4me3 and H3K27ac prediction in the validation NBL dataset with 10 samples.

In the revision:

“Using the validation set, we compared the performance of the M2A framework of three CNN layers and two FC layers (Figure 2) with three frequently used statistical and machine learning approaches (baseline models), namely multivariate adaptive regression splines (MARS), random forest, and artificial neural network (ANN) consisting of only two FC layers. In every instance, the M2A framework outperformed baseline models (Additional file 2: Table S2). From a qualitative perspective, M2A correctly revealed the bimodal distribution of the promoter activities for both H3K4me3 and H3K27ac in all samples, and from a quantitative perspective, the inferred genome-wide promoter activity landscape was highly accurate for individual samples for both H3K4me3 (*R*^2^ = 0.933 ± 0.019; RMSE = 0.621 ± 0.072) (Figures 3a and 3d) and H3K27ac (*R*^2^ = 0.799 ± 0.053; RMSE = 0.644 ± 0.074) (Figures 3b, and 3c). Moreover, the addition of CNN layers was merited, as there was a decrease in the prediction error (measured as 1 − *R*^2^) from the next highest performer by 17.8% (*P* = 0.0020, Wilcoxon signed-rank test) and 12.4% (*P* = 0.0020, Wilcoxon signed-rank test) for the model topologies for H3K4me3 and H3K27ac, respectively (Additional file 2: Table S2).”

4) M2A is based solely on the methylation data. Can the DNA sequence data be leveraged to increase the prediction accuracy?

**Response:** We consider DNA sequence-based features as static features, which are shared among different samples for the same promoter. In over-parameterized deep-learning models, including such static features raises the concern of model overfitting by potentially memorizing the identity/genomic position of the promoter (considering all training samples came from a single disease). Indeed, we found that including an additional static feature (number of CpGs in each window) actually decreased the prediction accuracy in validation samples using baseline models. Moreover, a recent study (Xiao et al., 2020) demonstrated that 1) local epigenomic features are more informative (predictive) than local genomic features in predicting enhancer-promoter interactions using CNN models; and 2) integrating local genomic features into epigenomic features does not produce much predictive gain. Therefore, we decide not to include DNA sequence features in our feature design.

Reference:

Xiao M, Zhuang Z, Pan W: Local Epigenomic Data are more Informative than Local Genome Sequence Data in Predicting Enhancer-Promoter Interactions Using Neural Networks. Genes. 2020, 11(1), 41. 10.3390/genes11010041.

**Major comment 5)**

5) Two HM markers were investigated in this study. How is the performance for other markers?

**Response:** We share Reviewer 3’s enthusiasm for understanding how predictive DNA methylation profiles are of HMs. H3K4me3 and H3K27ac are chosen as the response variable in the current study because they are the most common histone modifications in active promoters. Our preliminary analysis suggested that additional active histone marks with sharp peaks in promoter regions (i.e. H3K9ac) showed good prediction accuracy. On the other side, repressive HMs with broad domain profiles (i.e., H3K27me3 and H3K9me3) require different model design (including the feature window size, response window size, different network topology, etc.), which is one of our future directions.

6) The evaluation plan can be better illustrated by some flow chart.

**Response:** We thank the reviewer for this suggestion, and we have included the evaluation scheme.in Fig. 2.

**2^nd^ round**

**Reviewer 1**

We thank the authors for updating their manuscript and incorporating the reviewers' comments. In general the authors addressed all the concerns that were raised in the original submission, where they provided additional comparisons and detailed explanation of their method. In addition, the new figures and the corresponding legends are more informative

A minor concern in the revised manuscript is the comparison of obtained R^2 between studies, which highlights M2A's superior performance. Although the authors clearly demonstrate M2A's performance and cite the Karlic (2010) study which showed H3K27ac levels are accurate predictors of expression levels, I still feel these numbers are rather high and a comparison across studies on the raw R^2 values might not be that meaningful. As an example, the Karlic (2010) study showed an r = 0.72 Pearson's correlation, which gives an R^2 = 0.52 much smaller than the average R^2 = 0.68 the authors obtained using M2A. And actually on the revised manuscript where the authors compared with a RandomForest baseline model, this achieved a relatively comparable performance to M2A; and as a consequence an RF model would also provide a higher R^2 when comparing with existing studies. That is why some pre-processing steps, or normalisation of HM activity data might explain this discrepancy? I am not implying there is some issue in the authors' code, mostly trying to explain where the discrepancy might come and why direct comparison's might not be meaningful. Just to state again that the training here is performed on DNA methylation patterns which predict HM activity, and subsequently we just correlate the predicted activity with gene expression levels. As I stated in the first round of revisions, 'my main concern is that the way these results are present implicate that only from DNAm patterns one can predict not only all HM activity, but also the corresponding gene expression levels (and only from one HM), without the need to perform ChIP-seq'. I am a bit concerned that these claims might not be fully backed up by the results shown in the manuscript.

**Reviewer 2**

The authors have conscientiously and to a large extent satisfactorily responded to my review. However, particularly given their thoroughly described results in independent datasets that indicate finite false-positive and false-negative rates for finding active promoter regions, they really should add a paragraph in the Discussion section to describe a typical use case for their M2A package. For example, if a researcher is using a series of FFPE blocks to try and infer promoter activities from genome-wide DNA methylation data, and no other materials are available for the study, where would M2A fit in to their overall strategy?

**Reviewer 3**

The authors have addressed my comments sufficiently. I have no other comments.

**Author response**

We thank the authors for updating their manuscript and incorporating the reviewers' comments. In general the authors addressed all the concerns I raised in the original submission, where they provided additional comparisons and detailed explanation of their method. In addition, the new figures and the corresponding legends are more informative.

Response: We thank the reviewer for their positive comments and appreciate that their concerns were addressed in the revised manuscript.

Minor Comment

1)A minor concern in the revised manuscript is the comparison of obtained R^2 between studies, which highlights M2A's superior performance. Although the authors clearly demonstrate M2A's performance and cite the Karlic (2010) study which showed H3K27ac levels are accurate predictors of expression levels, I still feel these numbers are rather high and a comparison across studies on the raw R^2 values might not be that meaningful. As an example, the Karlic (2010) study showed an r = 0.72 Pearson's correlation, which gives an R^2 = 0.52 much smaller than the average R^2 = 0.68 the authors obtained using M2A. And actually on the revised manuscript where the authors compared with a RandomForest baseline model, this achieved a relatively comparable performance to M2A; and as a consequence an RF model would also provide a higher R^2 when comparing with existing studies. That is why some pre-processing steps, or normalisation of HM activity data might explain this discrepancy? I am not implying there is some issue in the authors' code, mostly trying to explain where the discrepancy might come and why direct comparison's might not be meaningful. Just to state again that the training here is performed on DNA methylation patterns which predict HM activity, and subsequently we just correlate the predicted activity with gene expression levels. As I stated in the first round of revisions, 'my main concern is that the way these results are present implicate that only from DNAm patterns one can predict not only all HM activity, but also the corresponding gene expression levels (and only from one HM), without the need to perform ChIP-seq'. I am a bit concerned that these claims might not be fully backed up by the results shown in the manuscript.

Response: We thank the reviewer for the feedback and understand the concern that the Pearson’s R2 may not be easily comparable between different studies. Because we employed different analytical frameworks, we cannot rule out the possibility that pre-processing techniques also contribute to the apparent difference in R2. We have emphasized this potential caveat in the Discussion section.

In the revised manuscript:

“Strikingly, the correlation of M2A’s predicted promoter H3K27ac activities with gene expression levels across nine ENCODE cell lines (average R2 = 0.668) was comparable to the predictive accuracy of a model built on 11 HMs, one histone variant, and DNase I hypersensitivity [11]. Although these results are remarkable, we caution that differences in feature design and pre-processing steps (e.g. normalization of HM activity data) may contribute to the apparent high correlation with gene expression levels.”

Reviewer 2.

The authors have conscientiously and to a large extent satisfactorily responded to my review.

Response: We thank the reviewer for his/her positive comment and are glad that our revised manuscript addressed the concerns.

Minor Comment

1)However, particularly given their thoroughly described results in independent datasets that indicate finite false-positive and false-negative rates for finding active promoter regions, they really should add a paragraph in the Discussion section to describe a typical use case for their M2A package. For example, if a researcher is using a series of FFPE blocks to try and infer promoter activities from genome-wide DNA methylation data, and no other materials are available for the study, where would M2A fit in to their overall strategy?

Response: We wholeheartedly agree with Reviewer 2 and added an additional paragraph to the Discussion indicating how M2A can be uniquely useful, especially to researchers with FFPE samples testing hypotheses about epigenetic deregulation.

In the revised manuscript:

“Methylation2Activity allows us to answer several questions that have been impossible to address previously. For example, landscapes of comprehensive epigenetic deregulations in promoter regions can be inferred in large primary patient tumor cohorts preserved in FFPE, which previously has been intractable due to technical and financial challenges. The M2A inferred promoter activity landscape will greatly increase sample size, allowing robust statistical analysis to identify recurrent epigenetic deregulations in promoters.”
